# Use of Integrated Metabolic Maps as a Framework for Teaching Biochemical Pathways in the Pre-clinical Medical Curriculum

**DOI:** 10.1007/s40670-024-02073-1

**Published:** 2024-05-29

**Authors:** Kenny Nguyen, Jay R. Silveira, Karen M. Lounsbury

**Affiliations:** 1 The Office of Medical Education, The Robert Larner, MD College of Medicine, University of Vermont, Burlington, VT 05401 USA; 2 Department of Biochemistry, The Robert Larner, MD College of Medicine, University of Vermont, Burlington, VT 05401 USA; 3 Department of Pharmacology, The Robert Larner, MD College of Medicine, University of Vermont, Burlington, VT 05401 USA

**Keywords:** Medical biochemistry, Metabolism, Active learning, Visual diagram, Integrated mapping

## Abstract

**Introduction:**

The Larner College of Medicine has steadily transitioned to primarily active learning-based instruction. Although evaluations praise session formats, students often highlight difficulties in synthesizing preparatory materials to integrate biochemical pathways. A student/faculty collaboration led to the development of interactive metabolic maps that illustrate pathways and link to a broader framework of metabolism.

**Methods:**

A review of the session materials identified relevant biochemical pathways, and for each pathway, we created a fillable visual diagram to highlight the interactions between all substrates, enzymes, and cofactors. Implementation of the metabolic maps began for first-year medical students in fall 2022. Evaluation data included standard student session evaluations (Likert scale and qualitative comments) and a survey specific to the metabolic maps.

**Results:**

After implementing the maps, student ratings of biochemistry/metabolism session materials significantly improved (3.2 ± 1.04 to 4.3 ± 0.87, *p* < 0.001), and students made positive comments about their effectiveness. Most students (77.8%) used the metabolic maps to aid in studying biochemistry content for exams and found the metabolic maps important for integrating information about metabolic pathways. The median performance on metabolism-specific questions was higher, although not statistically significant (69.23 to 77.28, ns).

**Discussion:**

The implementation of integrated metabolic maps improved student satisfaction of biochemistry/metabolism session materials. Limitations include confounding factors related to student population differences and other simultaneous curriculum changes. Implementing interactive visual aids to integrate metabolism pathways and concepts is applicable to any medical curriculum, and other longitudinal topics may benefit from this type of curricular framework.

**Supplementary Information:**

The online version contains supplementary material available at 10.1007/s40670-024-02073-1.

## Introduction

There has been a growing body of evidence in recent years suggesting the benefits of active learning over traditional lecture-based teaching methods [1, 2]. These benefits are particularly relevant for medical education, where lecture-based pedagogy remains the primary teaching modality despite increasing focuses on collaboration and engagement within the healthcare field [3, 4]. As such, the Larner College of Medicine (LCOM) at the University of Vermont has gradually transitioned to a primarily active learning-based instruction. Whereas this transition has seen many improvements, including increased student participation in class and reception of session materials, students have highlighted difficulty in synthesizing preparatory materials for topics that integrate biochemistry pathways with direct and indirect effects on metabolism and pathophysiology. This difficulty is unfortunately a frequent challenge in medical education, with students often citing two primary obstacles to learning biochemistry in the context of lecture-based teaching methods: rote memorization of large amounts of factual information and difficulty understanding the interactions between different metabolic pathways and their relevance to clinical applications [5, 6].

Alternative methods of teaching biochemistry and metabolism have previously been shown to be effective, including in the usage of interactive maps and animation [7, 8]. Therefore, a student/faculty collaboration led to the development of interactive maps that visually illustrate metabolic pathways and link to a broader framework of metabolism. To create the maps, we reviewed previous session materials and identified relevant biochemical pathways. Both complete and blank, fillable visual diagrams were created for each pathway to showcase the interactions between all substrates, enzymes, and cofactors. We implemented the metabolic maps for biochemistry and metabolism instruction for first-year medical students in fall 2022 and 2023 and then used student evaluations and examination scores to gauge effectiveness. While the employment of metabolic maps to aid in biochemistry instruction is not a new concept [9–11], to our understanding, this manuscript is the first to offer a repository of easily downloadable and immediately usable interactive metabolic maps for the pre-clinical medical curriculum.

## Methods

We created twelve metabolic maps, shown in Online Resource 1 and 2, and PowerPoint versions to allow modification for other purposes are available from the corresponding author upon request. We chose vertical or horizontal layout depending on the feasibility and ease of visualizing particular pathways and their components.

The following vertically oriented maps (blank and completed) are found in Online Resource 1:

(A) Glycolysis
(B) Gluconeogenesis
(C) Fatty acid beta-oxidation and carnitine shuttle
(D) Fatty acid synthesis
(E) Ketone synthesis

The following horizontally oriented maps (blank and completed) are found in Online Resource 2:

(F) Primary fates of pyruvate
(G) Tricarboxylic acid (TCA) cycle
(H) Pentose phosphate pathway and hexose metabolism
(I) Electron transport chain
(J) Glycogenesis and glycogenolysis
(K) Methyl cycle
(L) Urea cycle

Evaluations/surveys used to collect student satisfaction data:

(M). Standard student session evaluation
(N). Specific metabolic map student survey

### Creation of Materials

We identified twelve essential biochemical pathways based on the metabolism concepts frequently tested on the United States Medical Licensing Examination (USMLE) Step 1 exam [12]. Each metabolic map showcased the interactions between all relevant substrates, enzymes, and cofactors. For each pathway, we created two versions of the map: a blank, fillable visual diagram and a nearly identical but full answer key.

We adapted information from the following resources for the creation of materials:

- Lippincott Illustrated Reviews: Biochemistry, 7th edition [13]
- Basic Medical Biochemistry: A Clinical Approach [14]
- Textbook of Biochemistry with Clinical Correlations, 7th edition [15]

Each metabolic map includes the following components:

- Pathway title labeled at the top left-hand corner
- Text boxes containing substrate names, representing each step in a pathway
- Directional or bidirectional arrows connecting two adjacent boxes, indicating reversible or irreversible reaction
- Dashed arrows indicating multiple steps (low yield enzymatic reactions on Step 1 and thus not included)
- Red arrows indicating allosteric inhibition
- Green arrows indicating allosteric stimulation
- Red asterisk indicating rate-limiting or committed step within the pathway
- Various colored shapes indicating coenzymes (e.g., CO_2_, ATP, NADH), where relevant
- Purple text indicating required vitamin coenzymes, where relevant

### Implementation

The goal of implementing the metabolic maps into first-year medical biochemistry workshops was to help students identify their strengths and knowledge gaps as they began each session and to build on existing knowledge as the concepts became more integrated in later sessions. Each metabolic map served as a component of the in-session learning materials for a single workshop activity in the Foundations of Clinical Science course, held in fall of the first-year. The assessments were primarily through readiness quizzes and 6 multiple choice block exams that covered 2–3 weeks of content. The medical biochemistry workshops were primarily delivered and assessed in blocks 2 and 3, but also integrated into other blocks and into later courses.

Students prepared for the workshops by independently reviewing pre-session preparatory materials (written documents, videos, and/or slide presentations) and taking a 10-question readiness quiz. Workshop sessions took place in a single large classroom and began by students assembling at tables in small groups (4–6 students) of their own choosing. Electronic versions of the blank metabolic maps were released to students at the start of the session, and since access to the files was not provided ahead of the workshop, this represented their first opportunity to work with the maps. Students then conversed with their group to fill in as much of the missing information on the maps as possible. Afterwards, the faculty instructor displayed the completed version of the map, discussed any areas of the map that the students in the session found especially challenging or confusing, and then presented application workshop questions that encouraged synthesis of the session’s metabolic concepts. The total scheduled workshop session time was 80 min. After the initial learning session, students were provided continuous access to the completed versions of the maps for review, and faculty in later courses revisited these maps for spaced repetition and layering of concepts applied to metabolic disorders.

### Modification and Usage of Maps

The twelve metabolic maps were either used, not used, or incorporated into integrated maps to supplement LCOM’s biochemistry/metabolism curriculum for first-year medical students. The included data represents student satisfaction, usage, and exam performance based on implementation of these adjusted maps. The following maps were used directly with minimal modification: (B) gluconeogenesis, (H) pentose phosphate pathway and hexose metabolism, (I) electron transport chain, and (J) glycogenesis and glycogenolysis. The following maps were modified or integrated together prior to use: (C) fatty acid beta-oxidation and carnitine shuttle, (D) fatty acid synthesis, (E) ketone synthesis, (G) tricarboxylic (TCA) cycle, (K) methyl cycle, and (L) urea cycle. The following maps were not used as part of the data, as they are planned to be used for other courses: (A) glycolysis and (F) formation of the four products of pyruvate.

### Evaluations

After each block exam, the 124 students submit confidential evaluations of individual sessions. To ensure all sessions are evaluated, but reduce survey burden, half of the class (62 students) is assigned to evaluate each session. We compiled responses for biochemistry sessions held prior to implementation (fall 2020 and 2021), and after implementation in fall 2022. In fall 2023, an additional short optional survey gathered feedback specific to the metabolic maps (124 students surveyed, 54 respondents). Evaluations included 5-point Likert scale ratings for both pre-session and session materials, and a section for general, open-ended comments (Online Resource 3). Statistical comparisons between groups were determined using unpaired Student’s *t* test. The specific survey asked if the metabolic maps help in studying and in integrating medical biochemistry using both Likert scale ratings and open-ended comments.

### Exam Performance

All exam questions link to session objectives and are grouped according to keywords; thus, we generated reports for the question performance as well as the student-specific performance on metabolism topics for fall 2021 and 2022. We compared specific performance on questions from block 2 exam due to its density of medical biochemistry sessions and the similarity of the workshops and exam questions before and after implementation. The maps presented and assessed in block 2 included the following: gluconeogenesis, pentose phosphate pathway and hexose metabolism, electron transport chain, and glycogenesis and glycogenolysis. Statistical comparisons between groups were determined using unpaired Student’s *t* test.

## Results

**Fig. 1:**
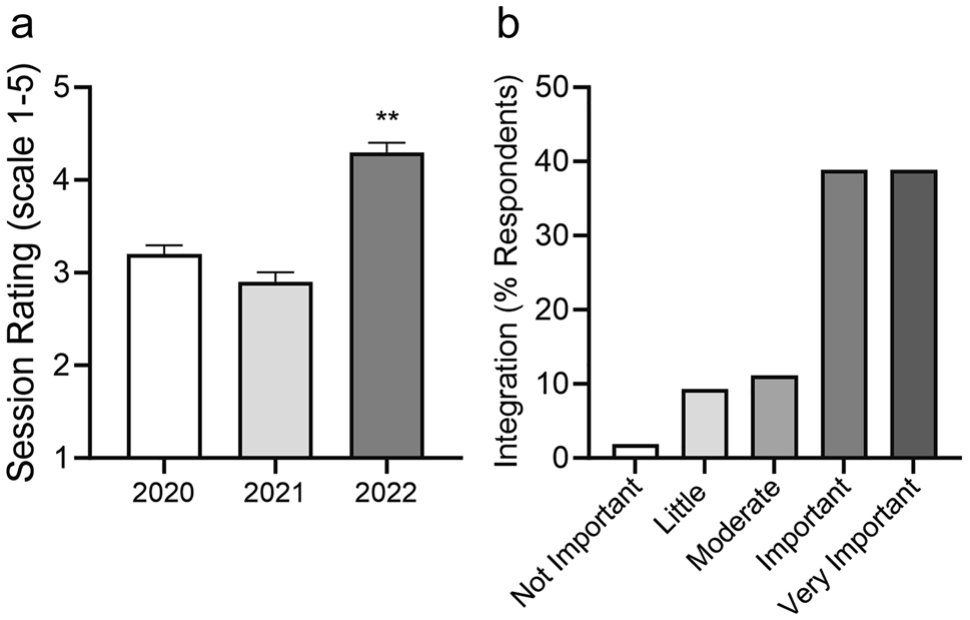
Ratings of biochemistry session materials and importance for integrating metabolism concepts. **a** Data were collected from session evaluations for the years indicated, and values represent the average Likert score ratings of specific biochemistry session materials in block 2. **p < 0.0001 by Student’s *t* test; *n* = 62–63 students/class. **b** Survey data were collected from students in fall 2023 regarding use and importance of the metabolic maps. Shown are % respondents in each of 5 importance categories, *n* = 54 students

We measured the effectiveness of the metabolic maps by comparing the student session evaluations before and after implementation (fall 2022). Students in fall 2022 rated the session materials 4.3 ± 0.87 on a 5-point Likert scale (*n* = 62), which was significantly improved compared to evaluations of the same session topic from fall 2020 (3.2 ± 1.04, *p* < 0.001; *n* = 62) and 2021 (2.9 ± 1.14, *p* < 0.001; *n* = 63) (Fig. 1a). In the open-ended feedback section from fall 2022, 20 students used the space to specifically comment on the usefulness of metabolic maps (Table 1). Eighteen of these comments were positive in nature, including “Beginning the workshops with a blank pathway that connects several aspects of the reading has been very helpful” and “I like the metabolic maps that we fill out and think they are helpful to link concepts and see things from a broad point of view.” Of these 18 comments, five additionally included recommendations to include the metabolic maps in the pre-session review materials. The remaining two comments voiced displeasure regarding the maps, including “I don’t find the metabolic maps particularly helpful.”

**Table 1:** Representative comments regarding metabolic maps and/or their usage from course evaluations in fall 2022

**Block number**^**a**^	**Student comments**
Block 2	“I appreciate that [the professor’s] workshops are always extremely engaging… The metabolic maps provided were helpful.”
	“I like the slides [the professor] has at the beginning of class where we use our prior knowledge/pre-work knowledge and try to fill out the map of the metabolic process. This helped me pinpoint what I already understand and what I need work on.”
	“The maps at the start of the workshops are really helpful but the prework word documents are very inaccessible for trying to understand the underlying concepts for the material.”
	“I really like the ‘fill in the blank’ mapping and the practice questions!”
	“The metabolic maps are awesome and I like the application questions.”
	“I like the metabolic maps that we fill out and think they are helpful to link concepts and see things from a broad point of view.”
	“Why not include those flow diagrams in our prework? It would conceptualize the material a lot better.”
	“I would suggest doing the flow chart as a part of the pre-work, it helps solidify the information.”
	“Beginning workshops with a blank pathway that connects several aspects of the reading has been very helpful.”
Block 3	“It would be helpful to have a longitudinal ‘map’ of all the metabolism pathways that we can build together as we learn different parts of the system throughout the block.”
	“The metabolism maps are very helpful.”
	“I really enjoy starting each class with the mapping exercise. They are extremely useful for studying afterwards.”
	“Appreciated… the concept mapping at the beginning of each session. Wish the mapping was moved to being part of the pre work though so we could spend more time reviewing in class.”
	“The metabolic maps were really helpful for glycolysis and TCA but as we’ve gotten into fatty acid oxidation and PPP they were confusing and I felt as though they were too in depth for what we need at this time.”
	“The maps tend to get confusing.”
	“Please include the metabolic maps as prework.”
	“It would be helpful to have the pre-filled metabolism map prior to workshop session to create a big picture understanding of the pathway.”
Block 4	“I like the way we can expand on prework in class with the mapping.”
Block 5	“I don’t find the metabolic maps particularly helpful.”
	“I also like the metabolic mapping at the start of each class session. Biochemical pathway maps help me to see the bigger picture of how everything interacts before I dive deeper into nuanced questions.”

^a^Each course block is 2–3 weeks in length. Student evaluations were obtained at the end of every block

In fall 2023, we distributed an optional three-question survey to first-year students (124 students) to specifically evaluate the newly implemented metabolic maps. Of the 54 students who completed the survey, 78% indicated they used the metabolic maps in studying for medical biochemistry content and that the maps had a positive impact on their ability to understand and integrate information about metabolic pathways (Fig. 1b). Students provided feedback on the benefits and disadvantages of using the metabolic maps during class and when studying for exams, including “I find them critical to piecing information together when studying” and “They are so complex that sometimes it’s easy to almost get lost in them” (Table 2).

**Table 2:** Qualitative comments from survey specific to metabolic maps

	**Number of comments**	**Example comment**
**Strengths**		
Visualization of pathway integration	13	“The maps help integrate our knowledge of how metabolic processes interconnect and are influenced by factors that spread throughout the body.”
An easy-to-comprehend visual format	9	“In my mind, metabolism is maps. So I look at the maps first, then seek out text explanations as needed.”
Self-quizzing modality	6	“I feel that one of the greatest advantages is filling in a blank metabolic map and having a filled-in one to check answers.”
**Areas for improvement**		
Overwhelming or chaotic	9	“The biggest downside is that the maps are so complex that sometimes it’s easy to get lost in them.”
Maps are not included within preparatory materials	7	“Doing the maps at the beginning of class isn’t always productive for me, since it’s the first time I’m being exposed to it and I often have to go back to my notes to fill it out.”
Includes too many details	3	“I would say sometimes the maps have a lot of information already in them, which can subconsciously hinder memorization.”
Not a useful in class activity	2	“I find that spending time to start a class filling out a metabolic map doesn’t increase my comprehension of the pathways as I haven’t understood the pathway to that level yet to fill one out.”

**Fig. 2:**
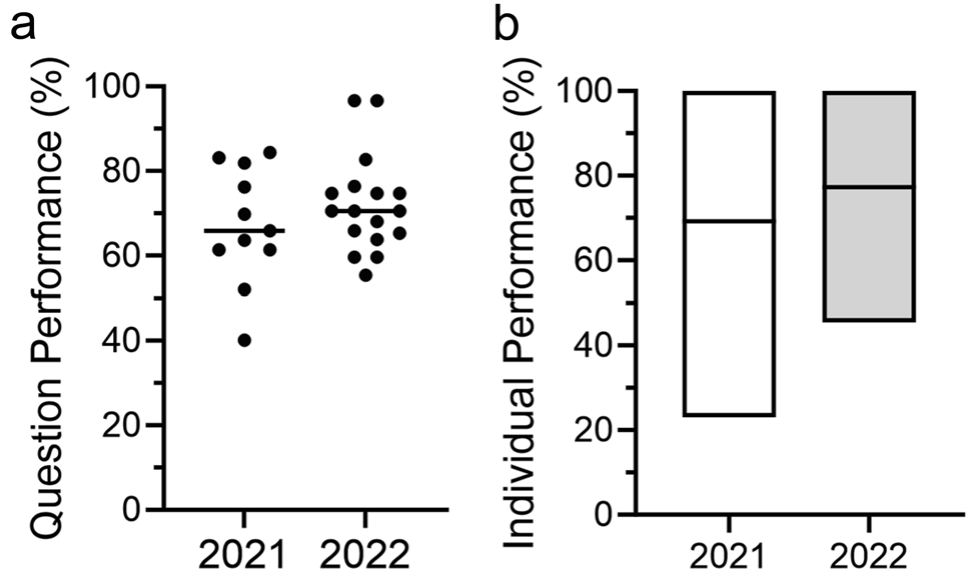
Student performance on biochemistry exam questions showed a positive, but not significant trend after implementing metabolic maps. **a** Points represent average student performance on individual questions and **b** boxes represent the 95% confidence interval for individual student performance. Questions mapped to biochemistry objectives in the year before vs. after implementing metabolic maps. Horizontal lines represent the median. Differences were not significant by unpaired Student’s *t* test

To determine if the maps had an impact on student performance on medical biochemistry exam questions, we stratified the questions that had objectives mapped to biochemistry/metabolism and compared the results from before (fall 2021) and after implementation (fall 2022). The median performance on individual questions trended higher but was not significantly different after implementation (65.90 to 70.50, ns) (Fig. 2a). When comparing individual student performance across metabolism-specific exam questions, the median was higher, and the performance range was smaller for fall 2022, but there was no overall significant difference in the groups (69.23 to 77.28, ns) (Fig. 2b). Together these data suggest that implementation of the metabolic maps improved student satisfaction, and while we observed a trend in improved student competency, the impact was too small to reach statistical significance in the data analysis.

## Discussion

The extensive positive feedback has led us to integrate the interactive metabolic maps into the ongoing biochemistry/metabolism curriculum for first-year medical students. To our knowledge, this report is the first to offer a repository of immediately usable biochemistry/metabolism visual maps for integration into the pre-clinical medical curriculum. The maps were created to represent and teach biochemistry/metabolism important to the USMLE Step 1 exam and thus may be easily implemented at any US medical institution with minimal adjustments necessary. The use of PowerPoint to create the maps allows interested users to edit the maps to fit their desired applications.

Per student session evaluations, the biochemistry instructors consistently received high ratings for their teaching, but session materials received lower scores prior the implementation of the maps. The majority of students from the fall 2023 cohort indicated that they used these maps to aid in studying for biochemistry/metabolism content on exams, and nearly all students found the maps important in integrating information about metabolic pathways. A few students made note of the “overwhelming” nature of the maps, but many students commented on the benefits, including in their utility to help visualize the larger-picture integration between individual metabolic pathways and use as a self-study tool (Table 2). Based on suggestions from several students, we will include the blank metabolic maps in the preparatory materials for the sessions as an additional benefit. Although the student satisfaction was higher, our study did not observe statistically significant differences in performance on medical biochemistry exam questions for first-year students that had access to the metabolic maps.

An increasing body of literature supports the complementary pairing of visual and interactive guides to traditional lecture for biochemistry/metabolism [9, 16], and previous studies accomplished success by utilizing metabolic maps [7] or 3D metabolism animation [8]. These studies varied in the style of supplemental material integration, with some providing the maps for study prior to lecture and others for use during class for group work. Regardless, both showed that a majority of students found the supplemental guides had a positive impact on their learning and understanding of biochemistry. Introducing the maps as part of the session can lead to some initial feelings of discomfort, since students may feel unprepared, and some students request the release of maps in advance. We balance the long-term benefit of this initial discomfort with overall satisfaction when the material is revisited in later courses. Similar to our findings, authors Gromley et al. [7] noted that some students found the metabolic maps to be “overwhelming” and “dense,” while authors Long et al. [8] mention students thought the animations did not include enough detail. This juxtaposition appears to suggest that the perceived “overwhelming” nature of metabolic maps can be reduced by standardizing the visual components and including links to additional information to reduce the density of visual information.

There are some potential confounding variables associated with the data collected for both session evaluations and exam performance due to the timeframe of our study. The instructors were different across the years, which may have affected the presentation of the biochemistry/metabolism objectives and the consistency of exam questions. Notably, we collected some of the evaluation and performance data from years when the COVID-19 pandemic affected the curriculum. We delivered the fall 2020 classes as hybrid online, and although the fall 2021 classes were in-person, exams did not return to in-person until fall 2022. Despite these factors, we observed a consistent increasing trend in student satisfaction that was supported by qualitative feedback after implementation of the maps.

Potential future avenues for this work include the creation of larger maps with animated layering to integrate individual metabolism pathways and the highlighting of pathway alterations that lead to pathologies. Doing so may allow students to more clearly visualize and understand the linkages between metabolic processes and various disorders/diseases rather than studying these molecular pathways in isolation. Creating similar interactive visual aids may also benefit the learning of other longitudinal topics in pre-clinical courses. In the session evaluations, students who used and found the maps helpful suggested creating similar visual aids for anatomy, a topic that shares several features with biochemistry, such as the need to rote memorize large amounts of information, which may contribute to difficulty in learning material under traditional lecture-based methods. Based on the continued success and elevated student satisfaction related to the metabolic maps, we hope to continue integrating visual aids in the pre-clinical curriculum to complement and support medical student education.

### Supplementary Information

Below is the link to the electronic supplementary material.

Supplementary file1 (PDF 709 KB)

Supplementary file2 (PDF 1494 KB)

Supplementary file3 (PDF 93 KB)

## Data Availability

Raw evaluation data analyzed during this study are not available in order to protect the anonymity of the student participants.
