# Supplementary material for: Use of Integrated Metabolic Maps as a Framework for Teaching Biochemical Pathways in the Pre-clinical Medical Curriculum
Source: Med Sci Educ. 2024 May 29;34(4):815–21. doi: 10.1007/s40670-024-02073-1 (PMC11296978; doi:10.1007/s40670-024-02073-1)
Supplement: Supplementary file 1 — Supplementary file1 (PDF 709 KB) [file 40670_2024_2073_MOESM1_ESM.pdf]

Metabolic Maps (Vertically Oriented)
Supplementary Online Resource 1

Article title: Use of Integrated Metabolic Maps as a Framework for Teaching Biochemical Pathways in the Pre-Clinical Medical Curriculum

Journal name: Medical Science Educator

Author names: Kenny Nguyen, Jay R. Silveira, Karen M. Lounsbury

Affiliation and email address of corresponding author:
The Robert Larner, MD College of Medicine, University of Vermont, Burlington, VT
karen.lounsbury@med.uvm.edu

GLYCOLYSIS

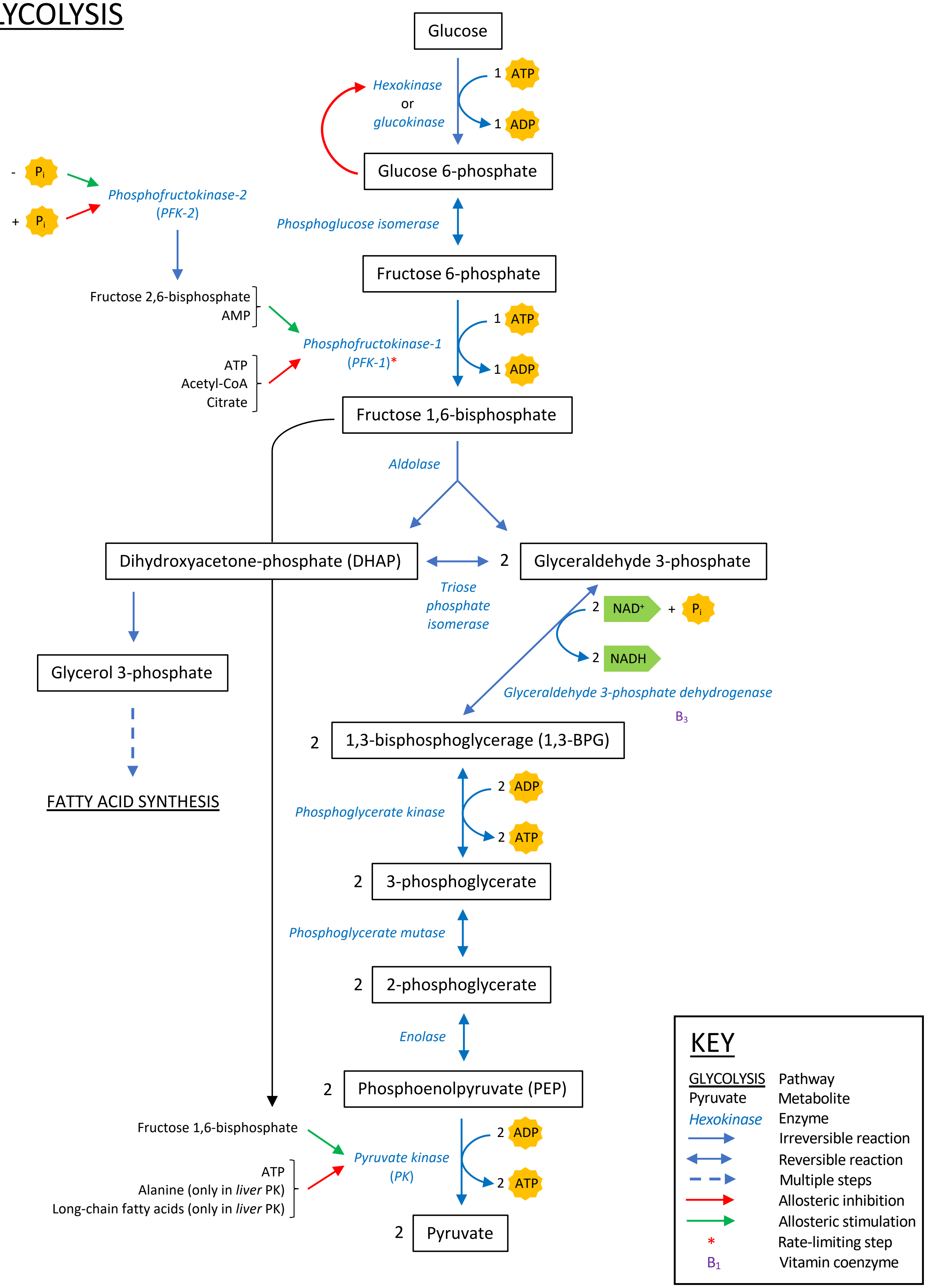

Glucose
Hexokinase
or
glucokinase
1 ATP
1 ADP
Glucose 6-phosphate
- P_i
+ P_i
Phosphofructokinase-2
(PFK-2)
Phosphoglucose isomerase
Fructose 6-phosphate
Fructose 2,6-bisphosphate
AMP
1 ATP
Phosphofructokinase-1
(PFK-1)*
1 ADP
ATP
Acetyl-CoA
Citrate
Fructose 1,6-bisphosphate
Aldolase
Dihydroxyacetone-phosphate (DHAP)
2 Glyceraldehyde 3-phosphate
Triose
phosphate
isomerase
2 NAD^+ + P_i
2 NADH
Glyceraldehyde 3-phosphate dehydrogenase
B_3
Glycerol 3-phosphate
FATTY ACID SYNTHESIS
2 1,3-bisphosphoglycerage (1,3-BPG)
2 ADP
Phosphoglycerate kinase
2 ATP
2 3-phosphoglycerate
Phosphoglycerate mutase
2 2-phosphoglycerate
Enolase
2 Phosphoenolpyruvate (PEP)
Fructose 1,6-bisphosphate
2 ADP
Pyruvate kinase
(PK)
ATP
Alanine (only in liver PK)
Long-chain fatty acids (only in liver PK)
2 ATP
2 Pyruvate
KEY
GLYCOLYSIS Pathway
Pyruvate Metabolite
Hexokinase Enzyme
Irreversible reaction
Reversible reaction
Multiple steps
Allosteric inhibition
Allosteric stimulation
* Rate-limiting step
B_1 Vitamin coenzyme

GLYCOLYSIS

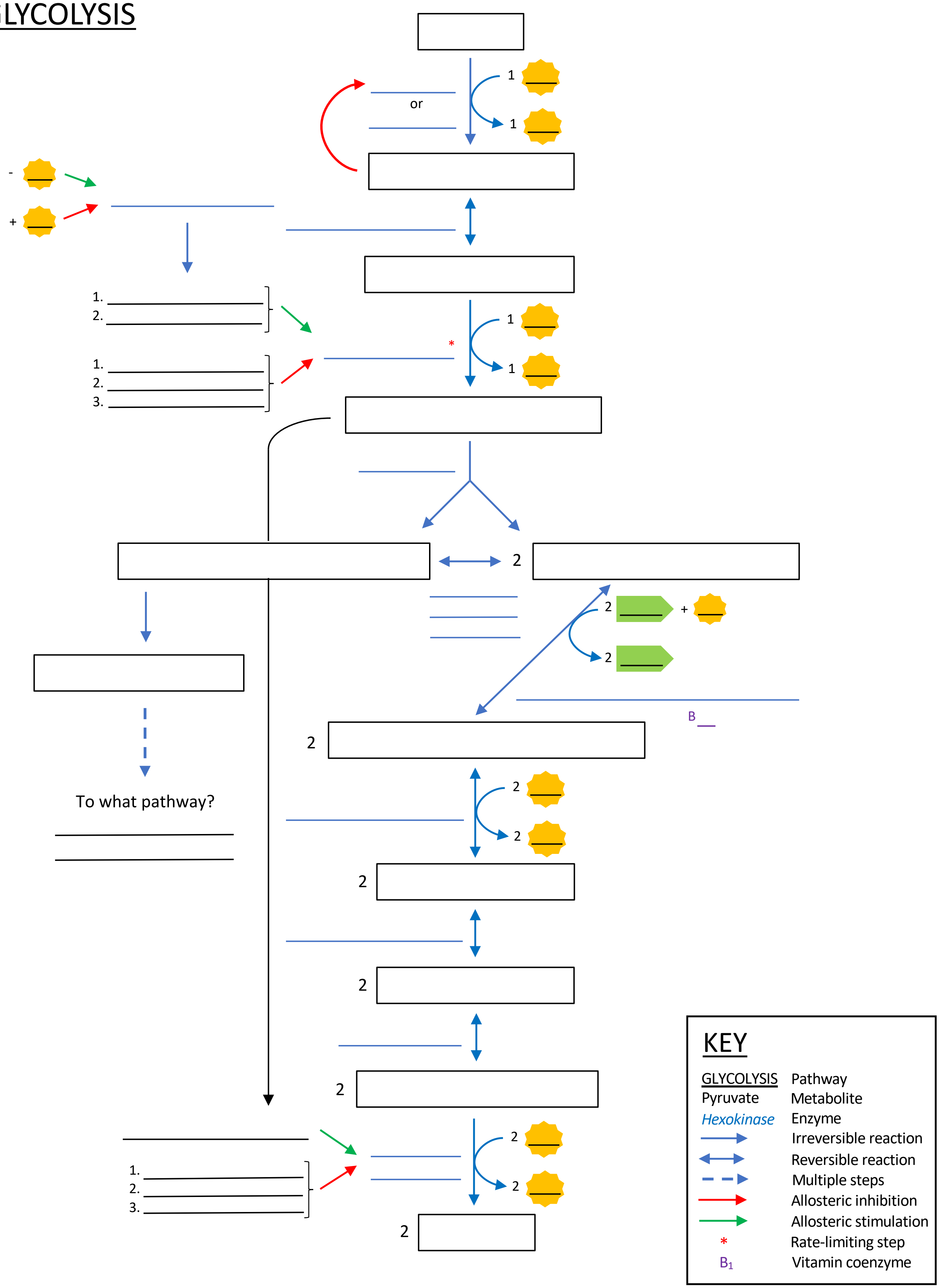

or
1
1
-
+
1.
2.
1.
2.
3.
*
2
2
+
2
B
2
To what pathway?
2
2
2
2
2
2
1.
2.
3.
2
2
KEY
GLYCOLYSIS Pathway
Pyruvate Metabolite
Hexokinase Enzyme
Irreversible reaction
Reversible reaction
Multiple steps
Allosteric inhibition
Allosteric stimulation
* Rate-limiting step
B1 Vitamin coenzyme

GLUCONEOGENESIS

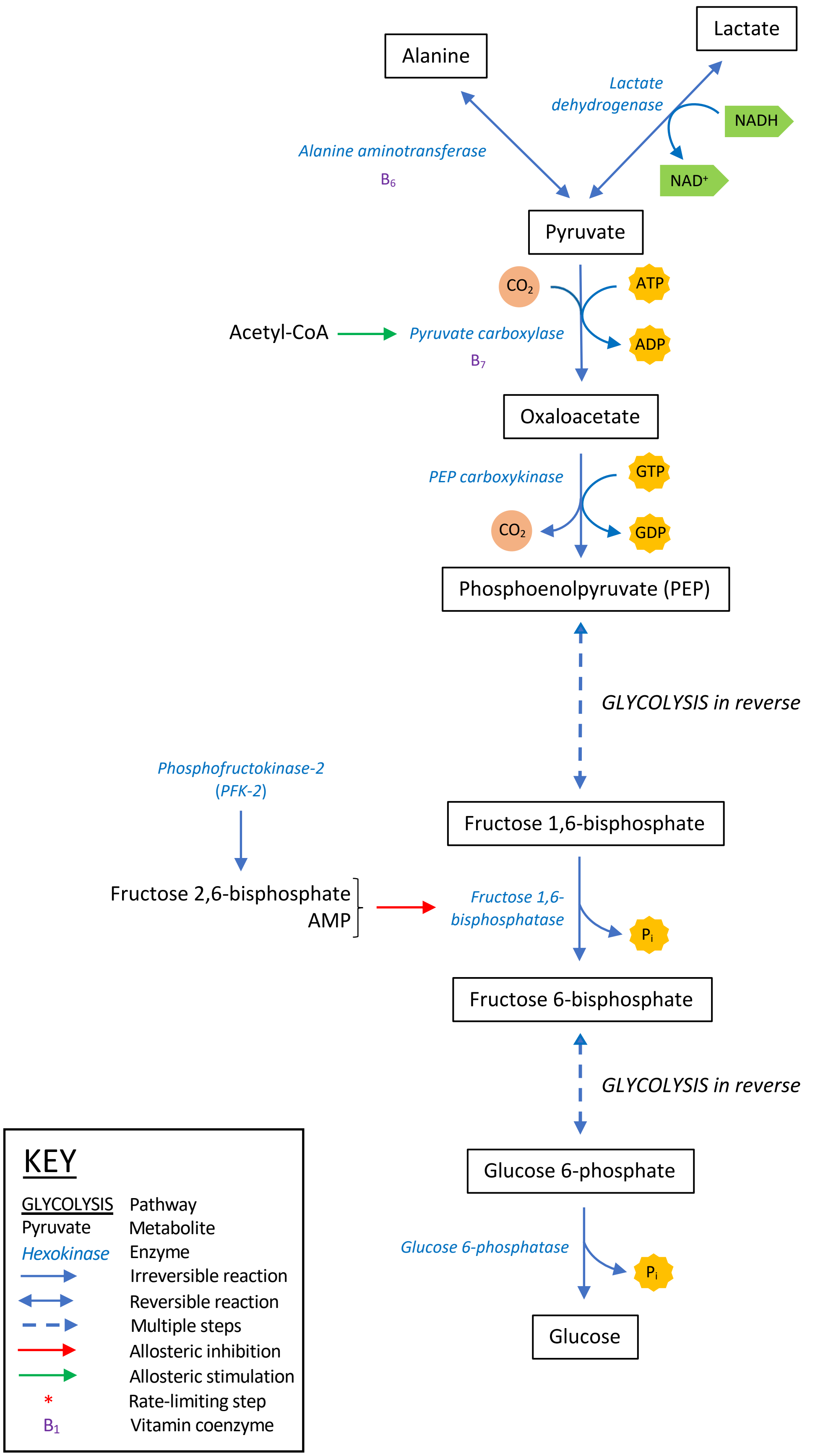

GLUCONEOGENESIS

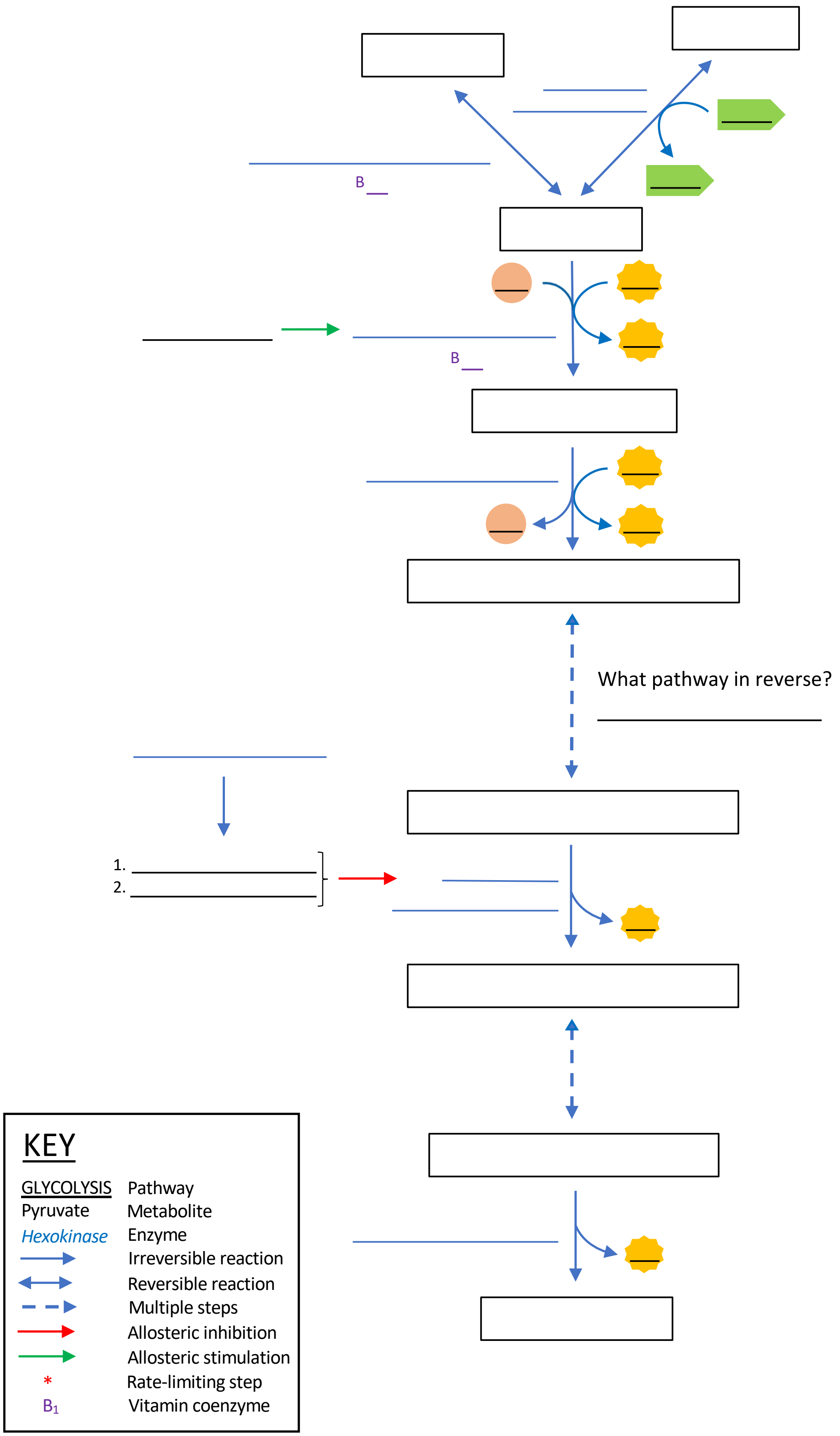

β-OXIDATION & CARNITINE SHUTTLE

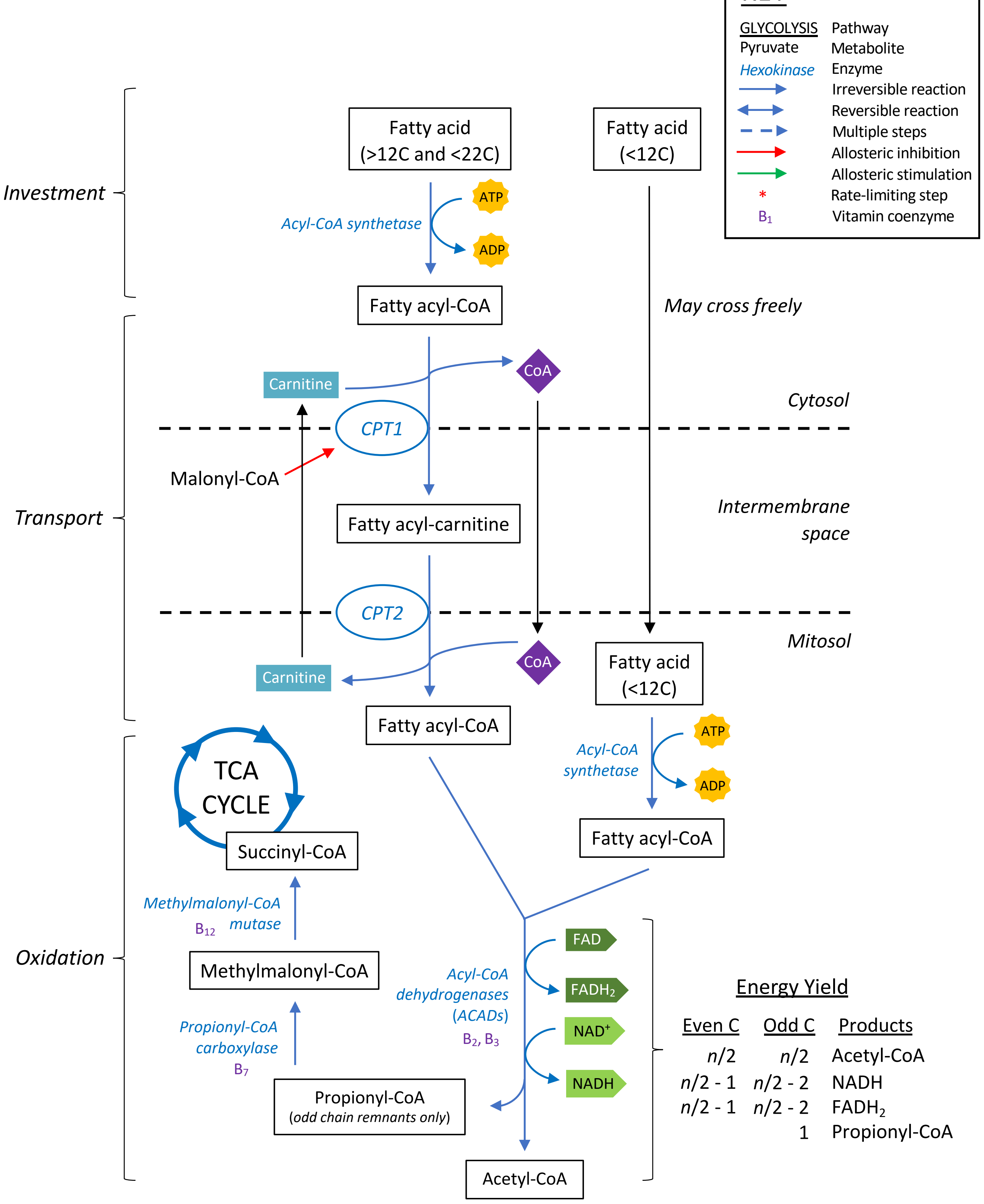

β-OXIDATION & CARNITINE SHUTTLE

KEY

GLYCOLYSIS	Pathway
Pyruvate	Metabolite
Hexokinase	Enzyme
→	Irreversible reaction
↔	Reversible reaction
- - →	Multiple steps
→ (red)	Allosteric inhibition
→ (green)	Allosteric stimulation
*	Rate-limiting step
B_1	Vitamin coenzyme

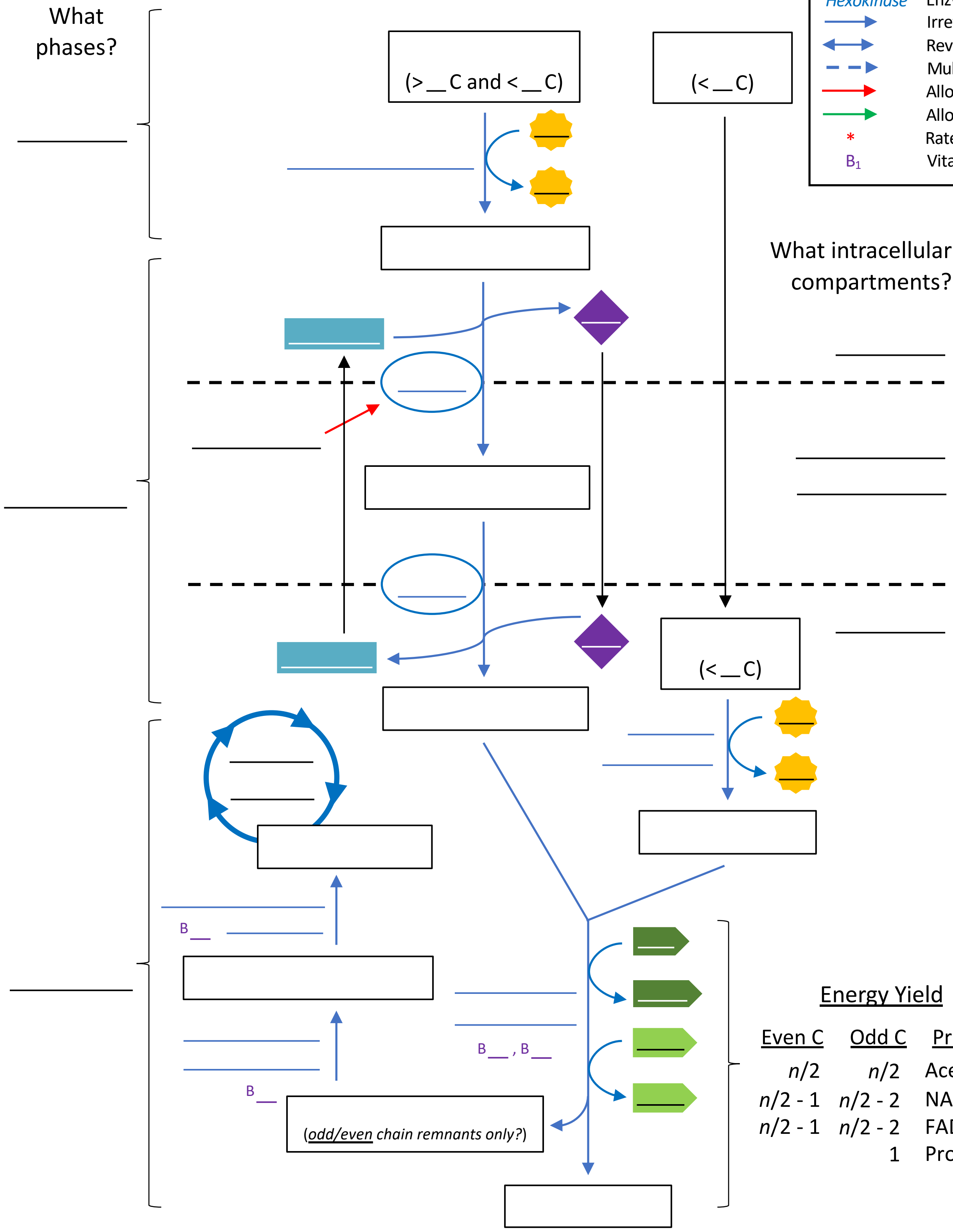

Energy Yield

Even C	Odd C	Products
$n/2$	$n/2$	Acetyl-CoA
$n/2 - 1$	$n/2 - 2$	NADH
$n/2 - 1$	$n/2 - 2$	$FADH_2$
	1	Propionyl-CoA

FATTY ACID SYNTHESIS

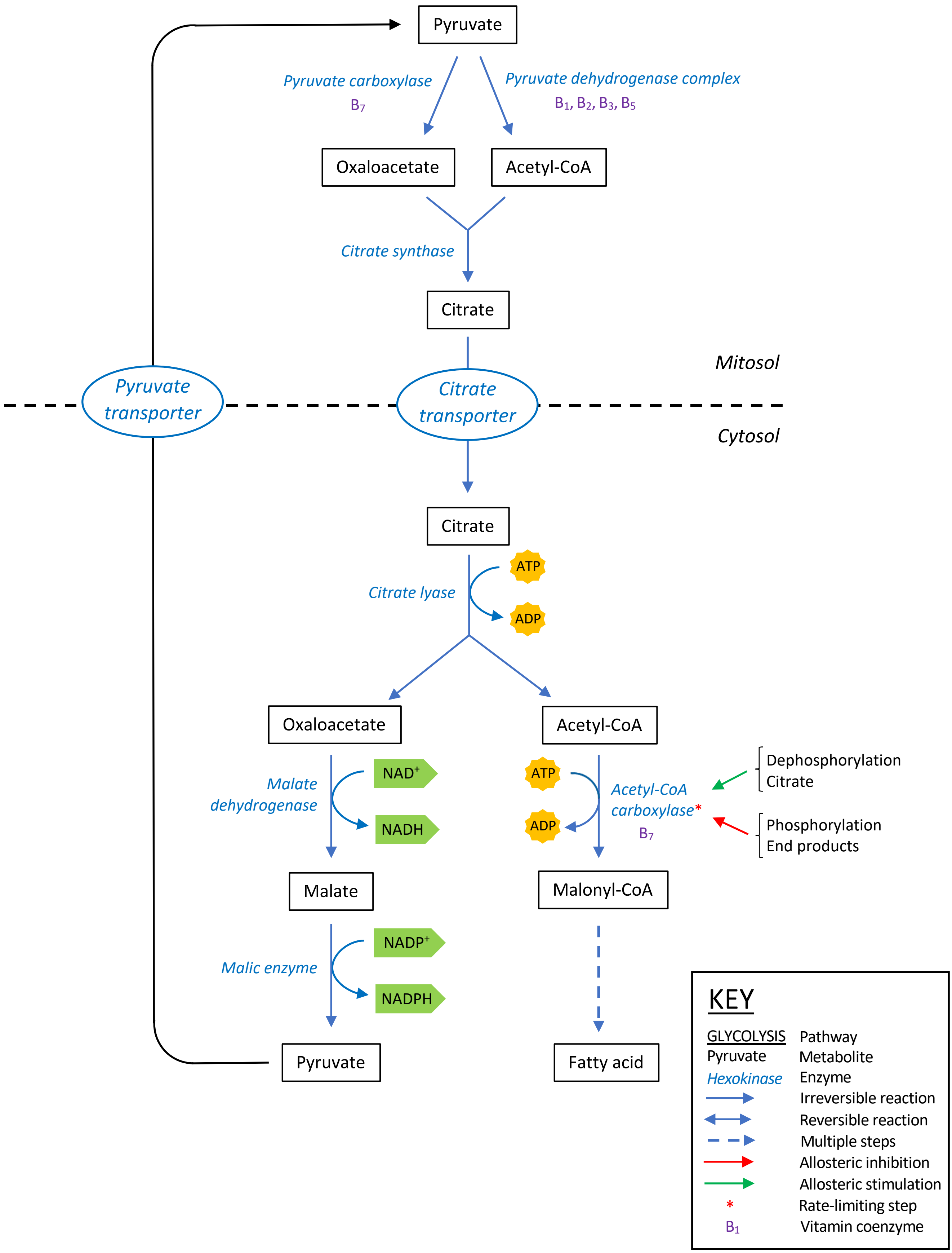

FATTY ACID SYNTHESIS

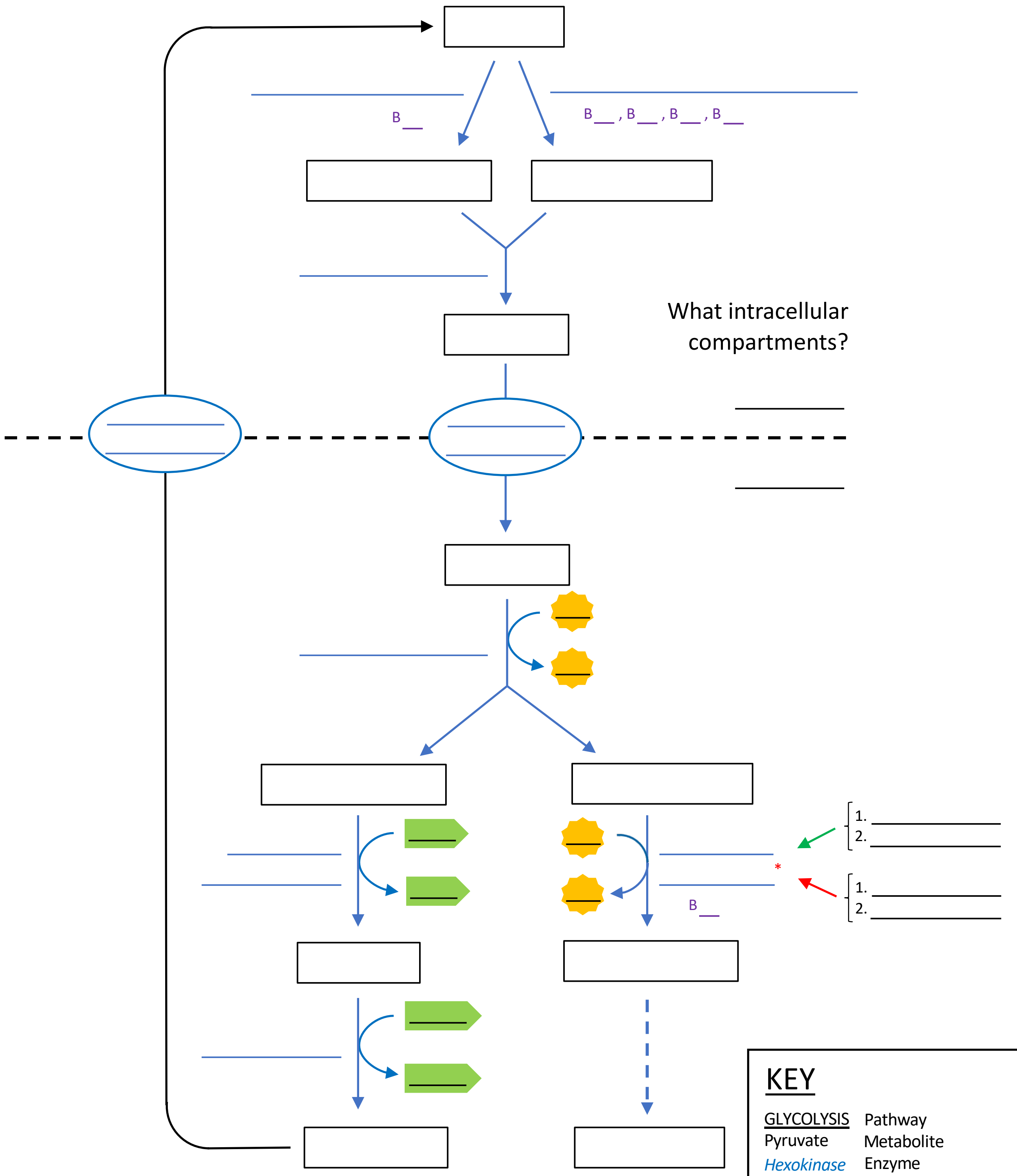

KEY

GLYCOLYSIS	Pathway
Pyruvate	Metabolite
Hexokinase	Enzyme
→	Irreversible reaction
↔	Reversible reaction
- - →	Multiple steps
→ (red)	Allosteric inhibition
→ (green)	Allosteric stimulation
*	Rate-limiting step
B_1	Vitamin coenzyme

KETONE SYNTHESIS

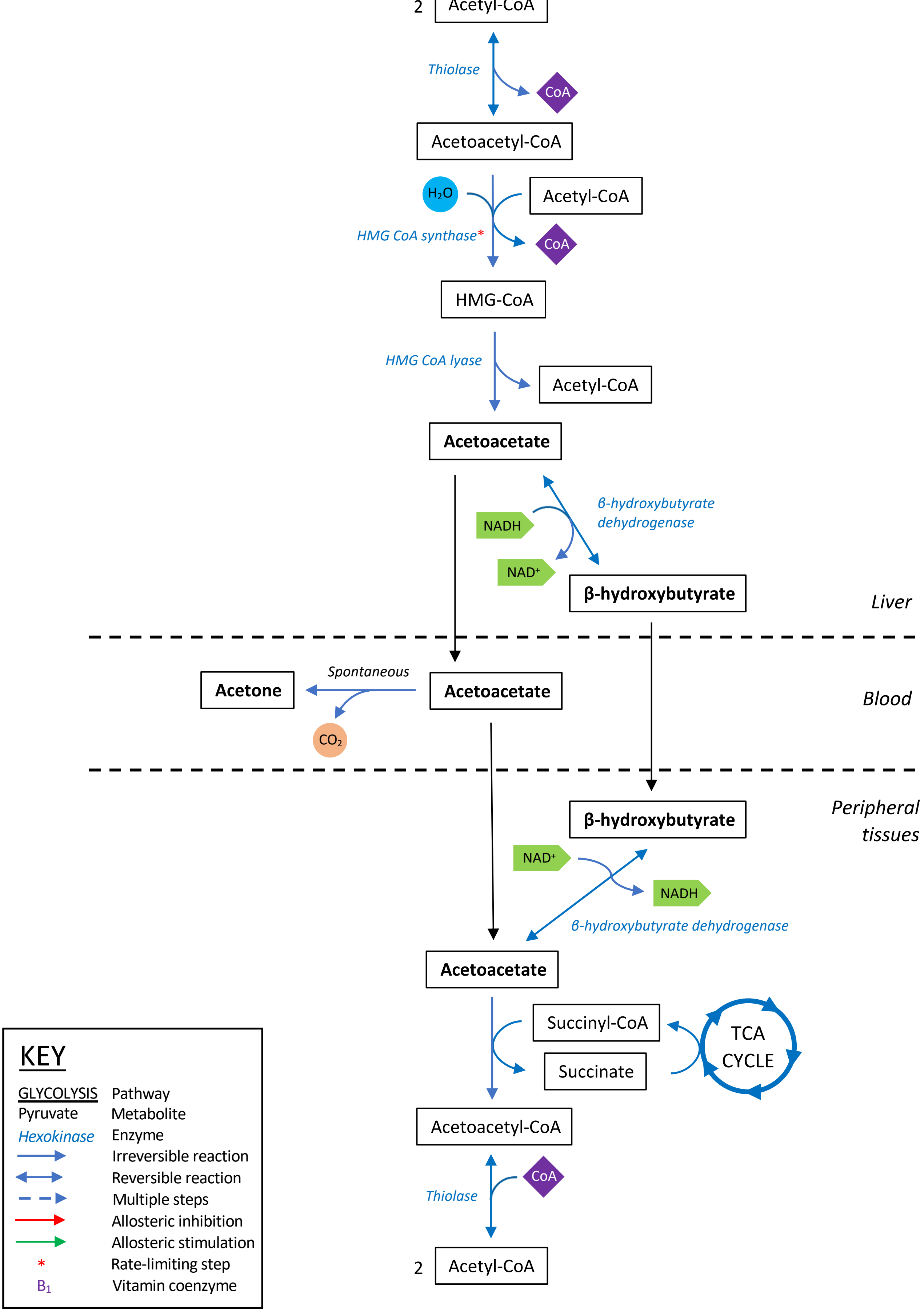

KETONE SYNTHESIS

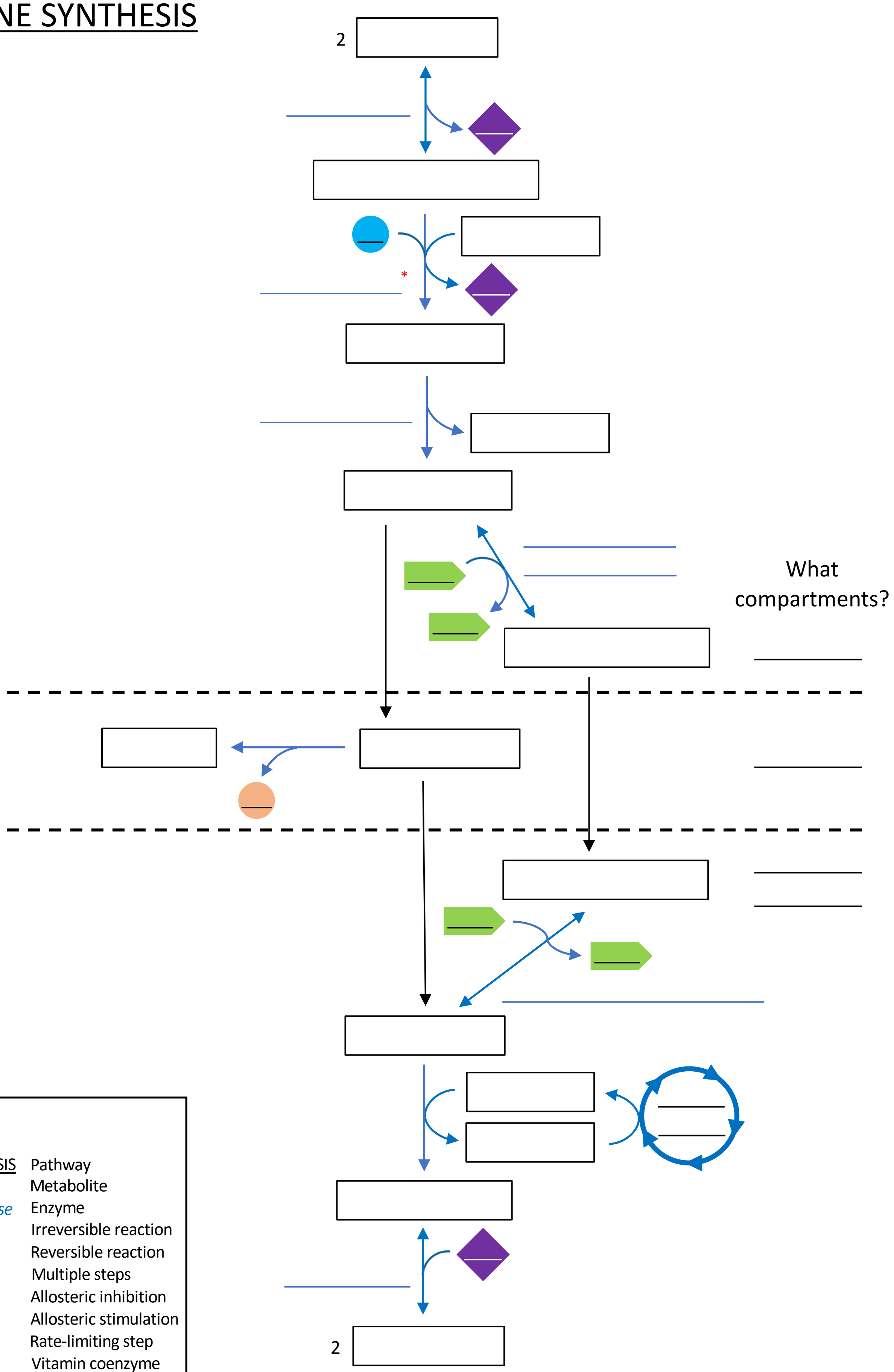

KEY

GLYCOLYSIS	Pathway
Pyruvate	Metabolite
Hexokinase	Enzyme
	Irreversible reaction
	Reversible reaction
	Multiple steps
	Allosteric inhibition
	Allosteric stimulation
*	Rate-limiting step
B_1	Vitamin coenzyme
