# Supplementary material for: Use of Integrated Metabolic Maps as a Framework for Teaching Biochemical Pathways in the Pre-clinical Medical Curriculum
Source: Med Sci Educ. 2024 May 29;34(4):815–21. doi: 10.1007/s40670-024-02073-1 (PMC11296978; doi:10.1007/s40670-024-02073-1)
Supplement: Supplementary file 2 — Supplementary file2 (PDF 1494 KB) [file 40670_2024_2073_MOESM2_ESM.pdf]

**Metabolic Maps (Horizontally Oriented)**
Supplementary Online Resource 2

**Article title:** Use of Integrated Metabolic Maps as a Framework for Teaching Biochemical Pathways in the Pre-Clinical Medical Curriculum

**Journal name:** Medical Science Educator

**Author names:** Kenny Nguyen, Jay R. Silveira, Karen M. Lounsbury

**Affiliation and email address of corresponding author:**
The Robert Larner, MD College of Medicine, University of Vermont, Burlington, VT
karen.lounsbury@med.uvm.edu

# PRIMARY FATES OF PYRUVATE

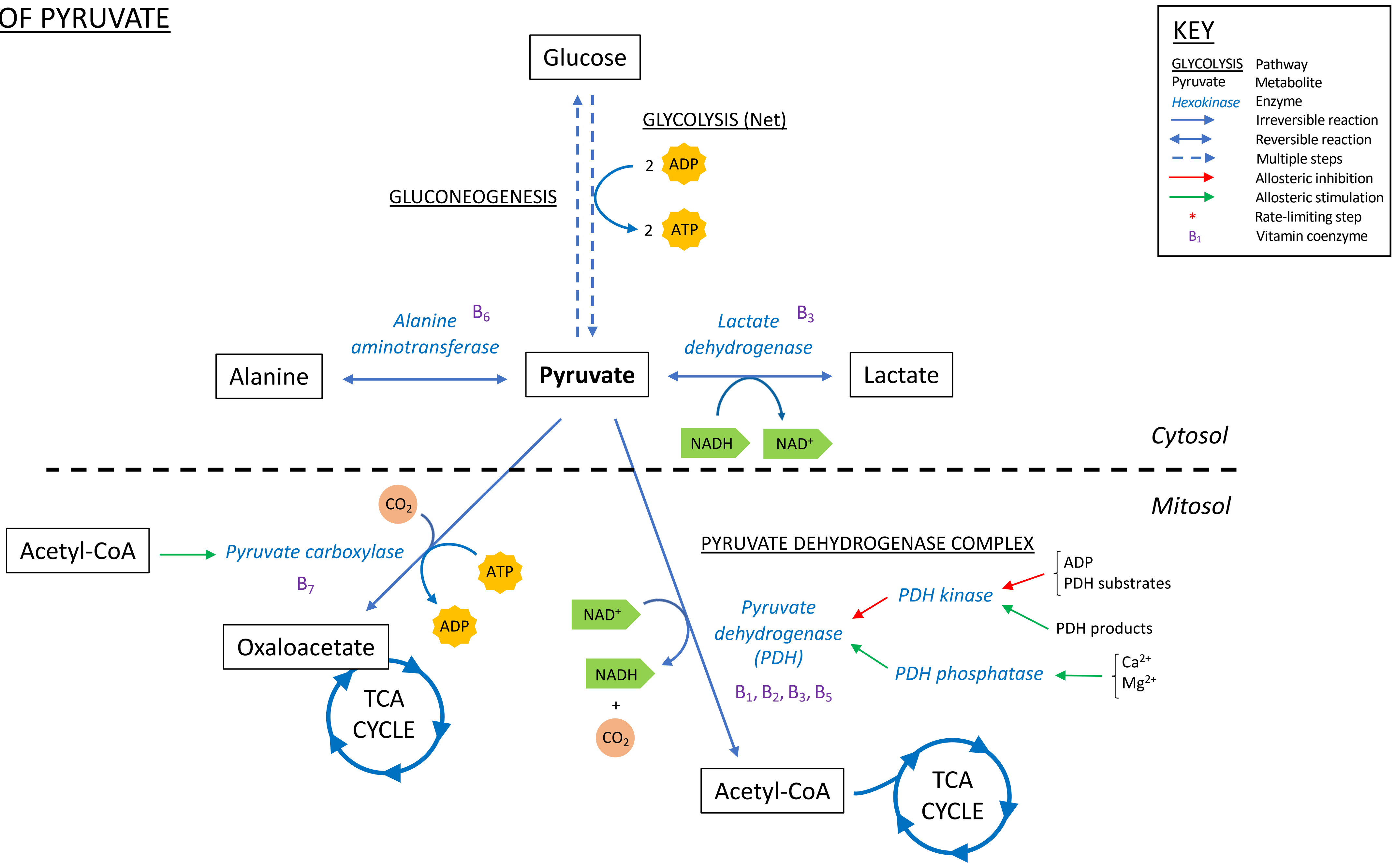

# PRIMARY FATES OF PYRUVATE

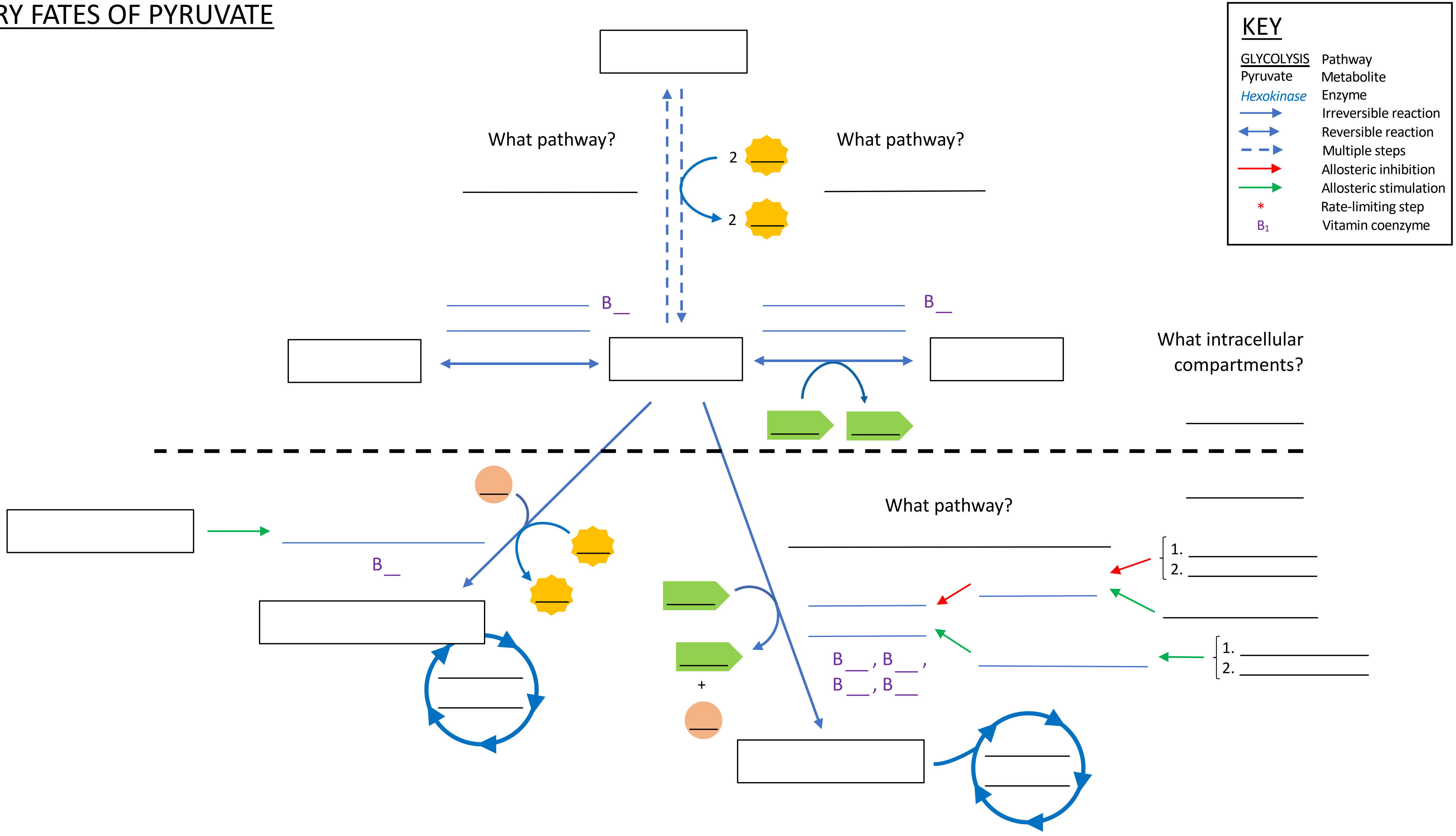

# TCA CYCLE

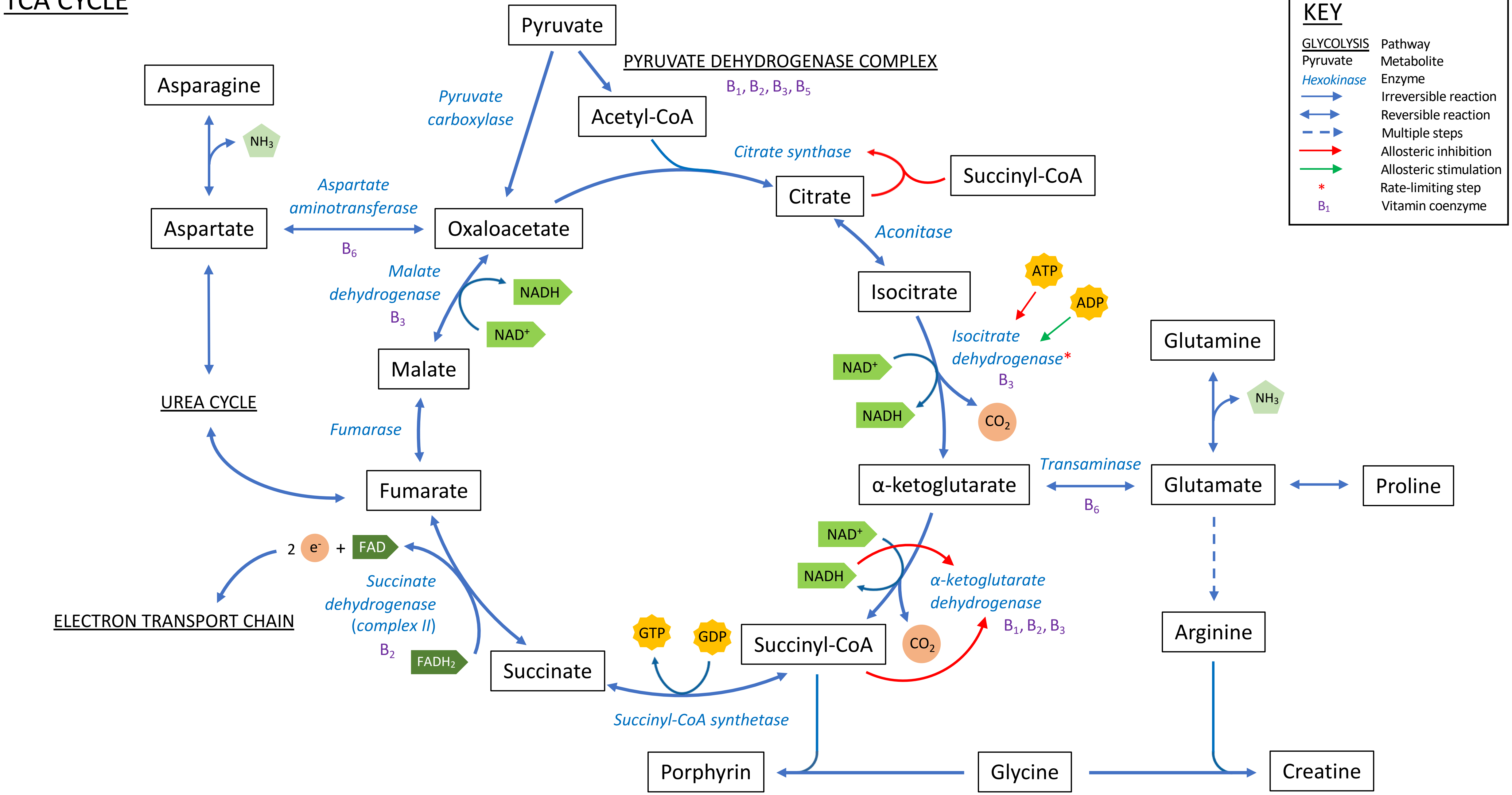

KEY
GLYCOLYSIS Pathway
Pyruvate Metabolite
Hexokinase Enzyme
Irreversible reaction
Reversible reaction
Multiple steps
Allosteric inhibition
Allosteric stimulation
* Rate-limiting step
$B_1$ Vitamin coenzyme
Pyruvate
PYRUVATE DEHYDROGENASE COMPLEX
$B_1$, $B_2$, $B_3$, $B_5$
Pyruvate carboxylase
Acetyl-CoA
Citrate synthase
Succinyl-CoA
Citrate
Aconitase
Isocitrate
ATP
ADP
Isocitrate dehydrogenase*
$B_3$
$NAD^+$
NADH
$CO_2$
α-ketoglutarate
Transaminase
$B_6$
Glutamine
$NH_3$
Glutamate
Proline
Arginine
$NAD^+$
NADH
α-ketoglutarate dehydrogenase
$B_1$, $B_2$, $B_3$
$CO_2$
Succinyl-CoA
GTP
GDP
Succinyl-CoA synthetase
Succinate
Succinate dehydrogenase (complex II)
$B_2$
$FADH_2$
2 $e^-$ + FAD
ELECTRON TRANSPORT CHAIN
Fumarate
Fumarase
Malate
Malate dehydrogenase
$B_3$
NADH
$NAD^+$
Oxaloacetate
Aspartate aminotransferase
$B_6$
Aspartate
Asparagine
$NH_3$
UREA CYCLE
Porphyrin
Glycine
Creatine

# TCA CYCLE

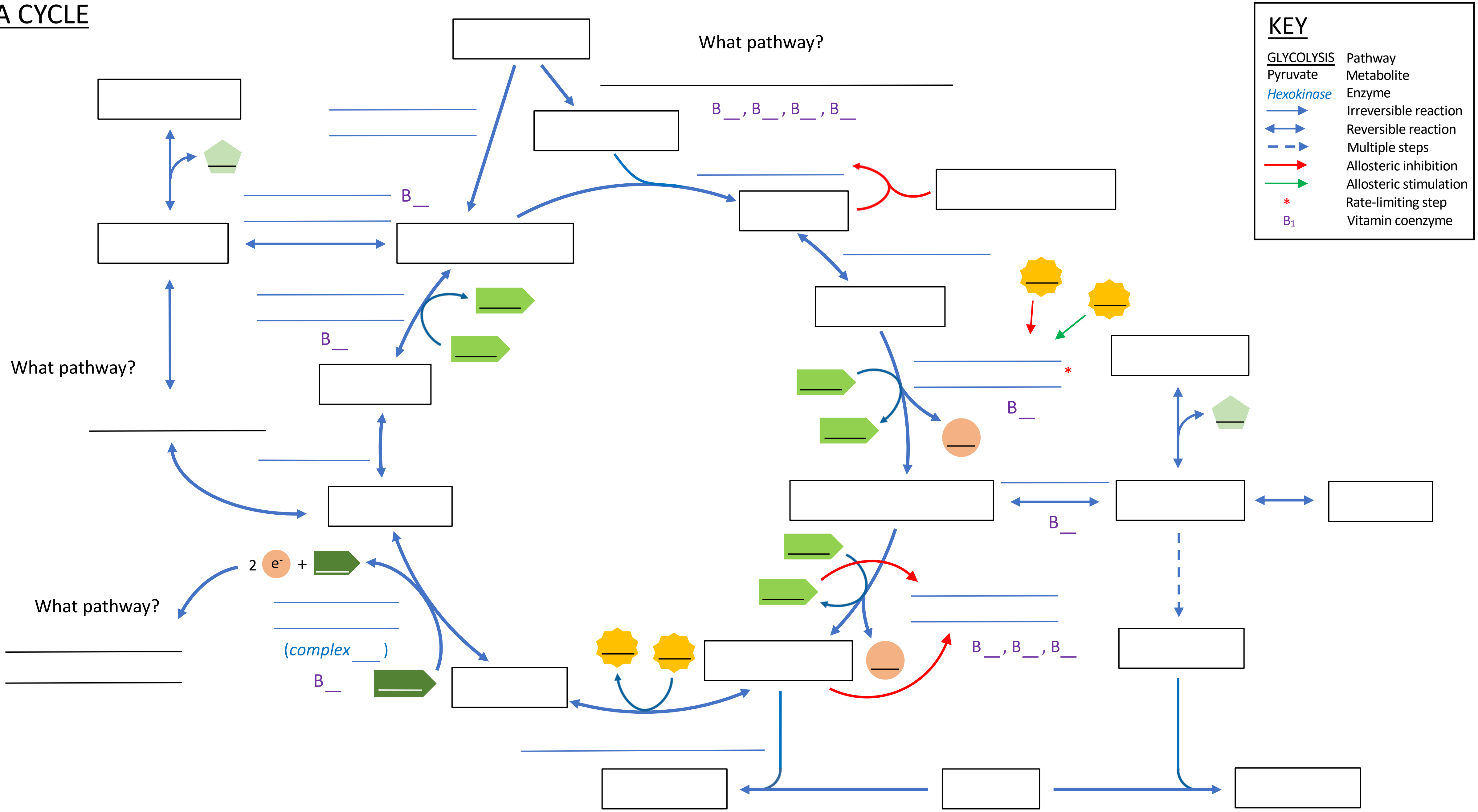

What pathway?
B__, B__, B__, B__
B__
B__
What pathway?
B__
B__
*
2 e- +
What pathway?
(complex__)
B__
B__, B__, B__
KEY
GLYCOLYSIS Pathway
Pyruvate Metabolite
Hexokinase Enzyme
Irreversible reaction
Reversible reaction
Multiple steps
Allosteric inhibition
Allosteric stimulation
* Rate-limiting step
$B_1$ Vitamin coenzyme

# PENTOSE AND HEXOSE METABOLISM

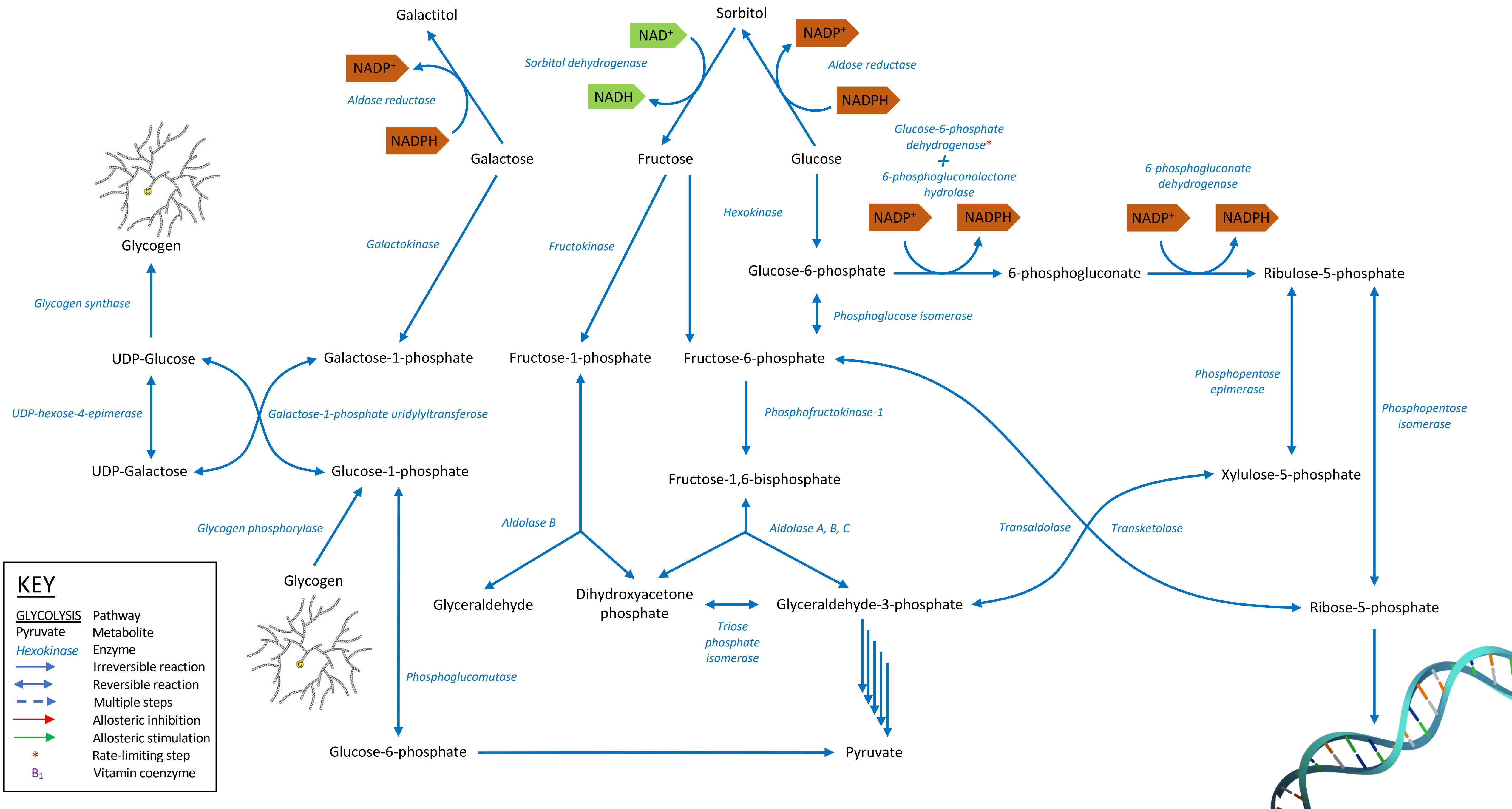

# PENTOSE AND HEXOSE METABOLISM

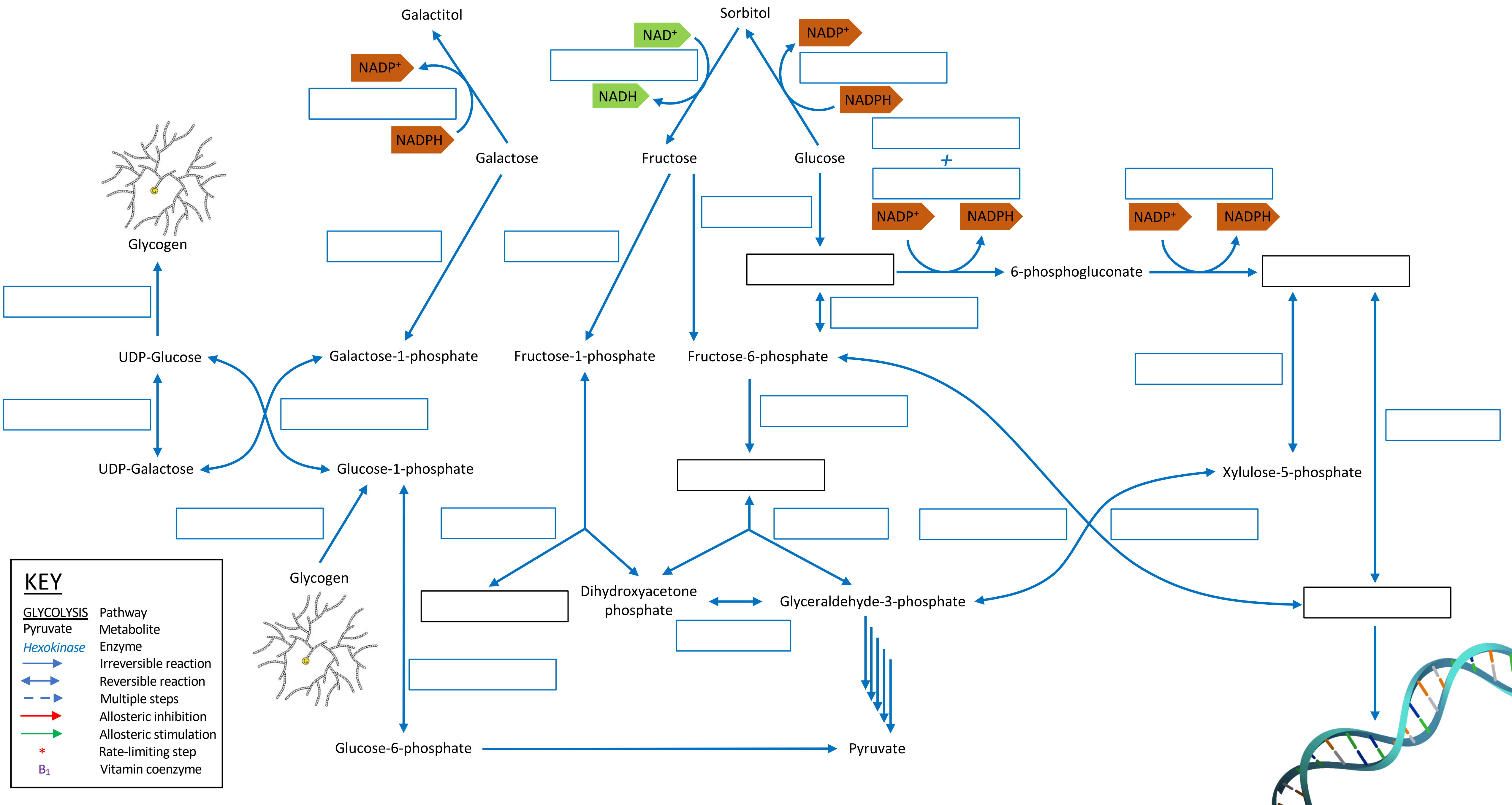

# ELECTRON TRANSPORT CHAIN

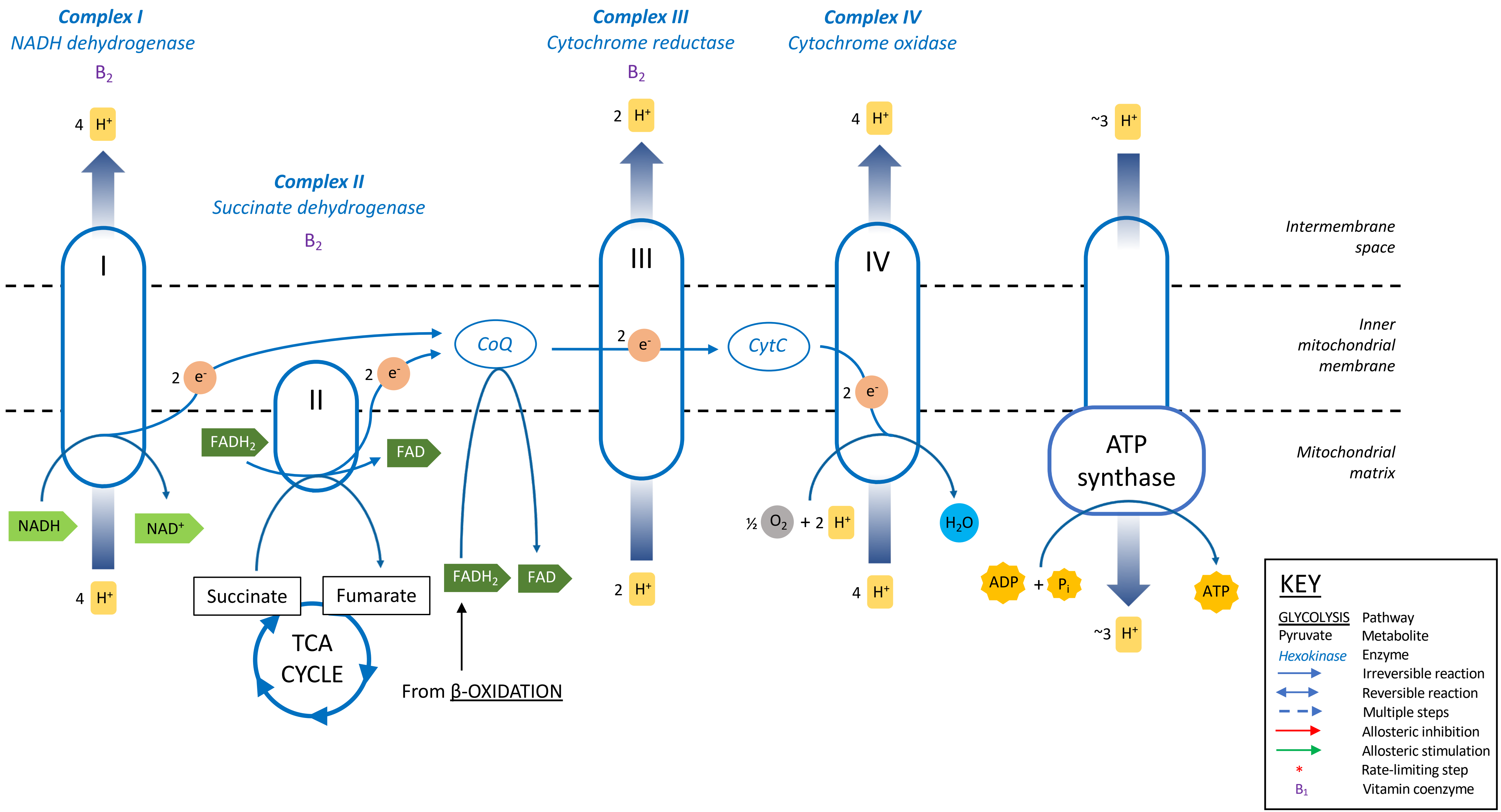

# ELECTRON TRANSPORT CHAIN

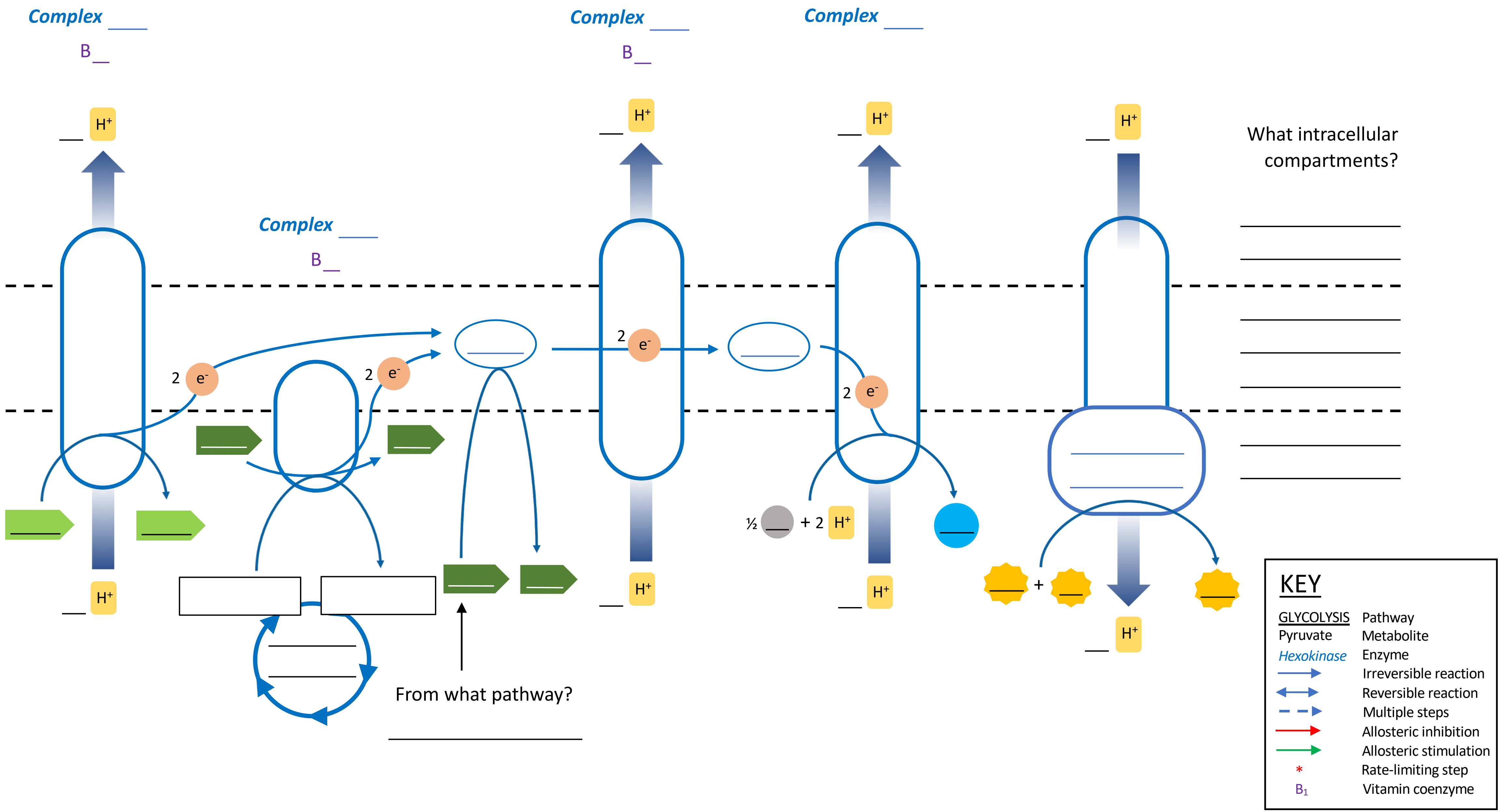

# GLYCOGENESIS & GLYCOGENOLYSIS

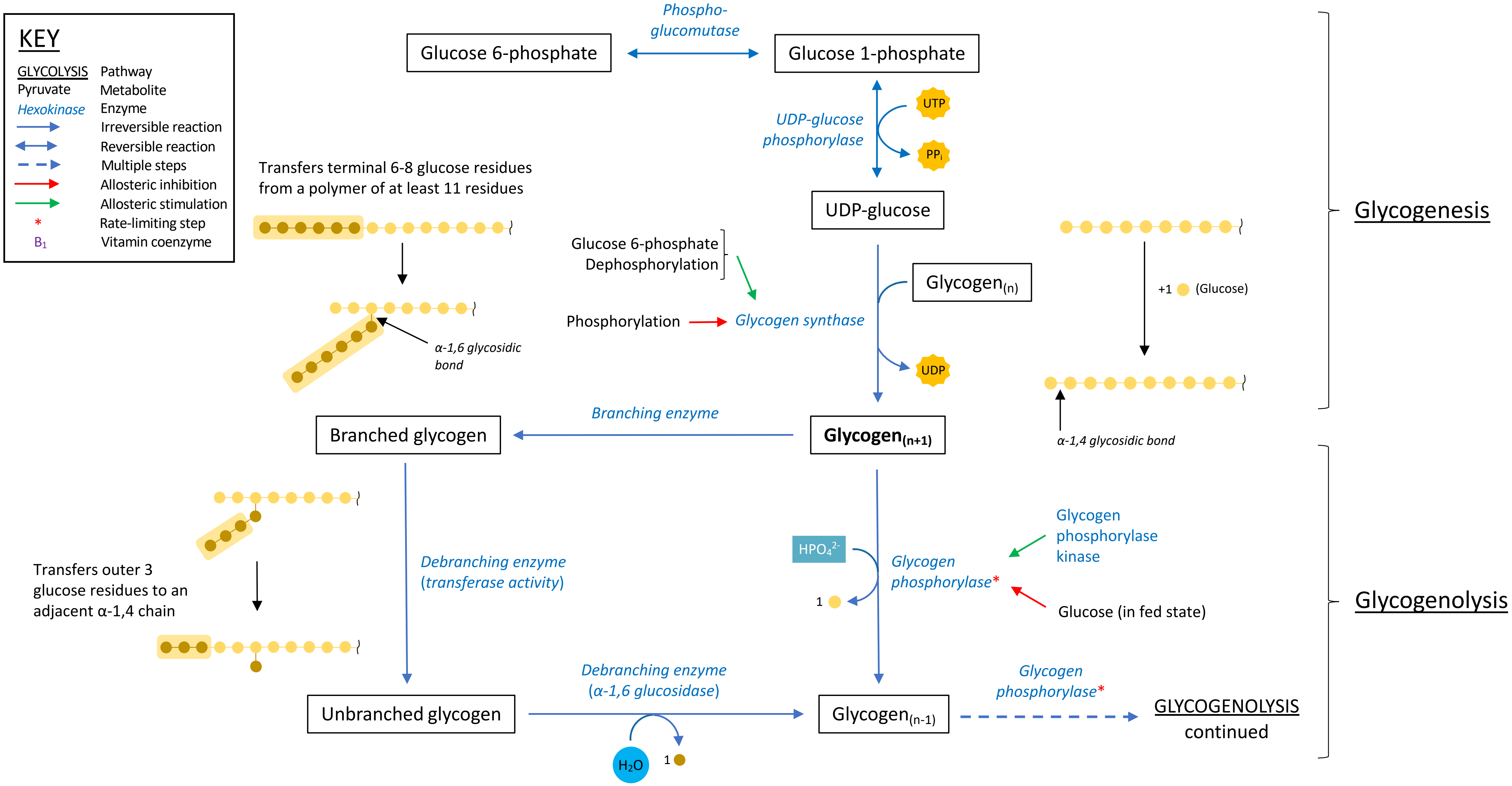

# GLYCOGENESIS & GLYCOGENOLYSIS

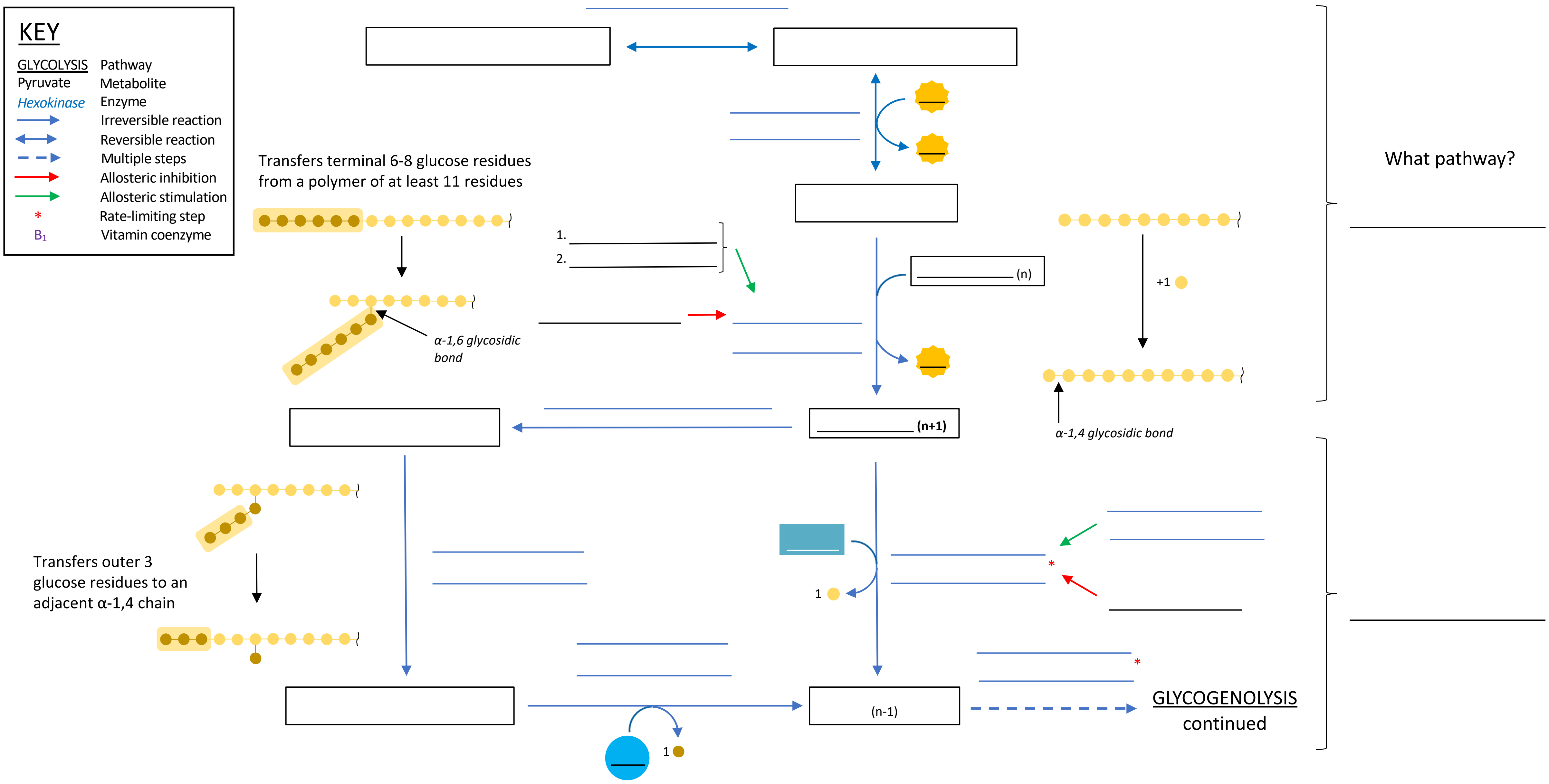

# METHYL CYCLE

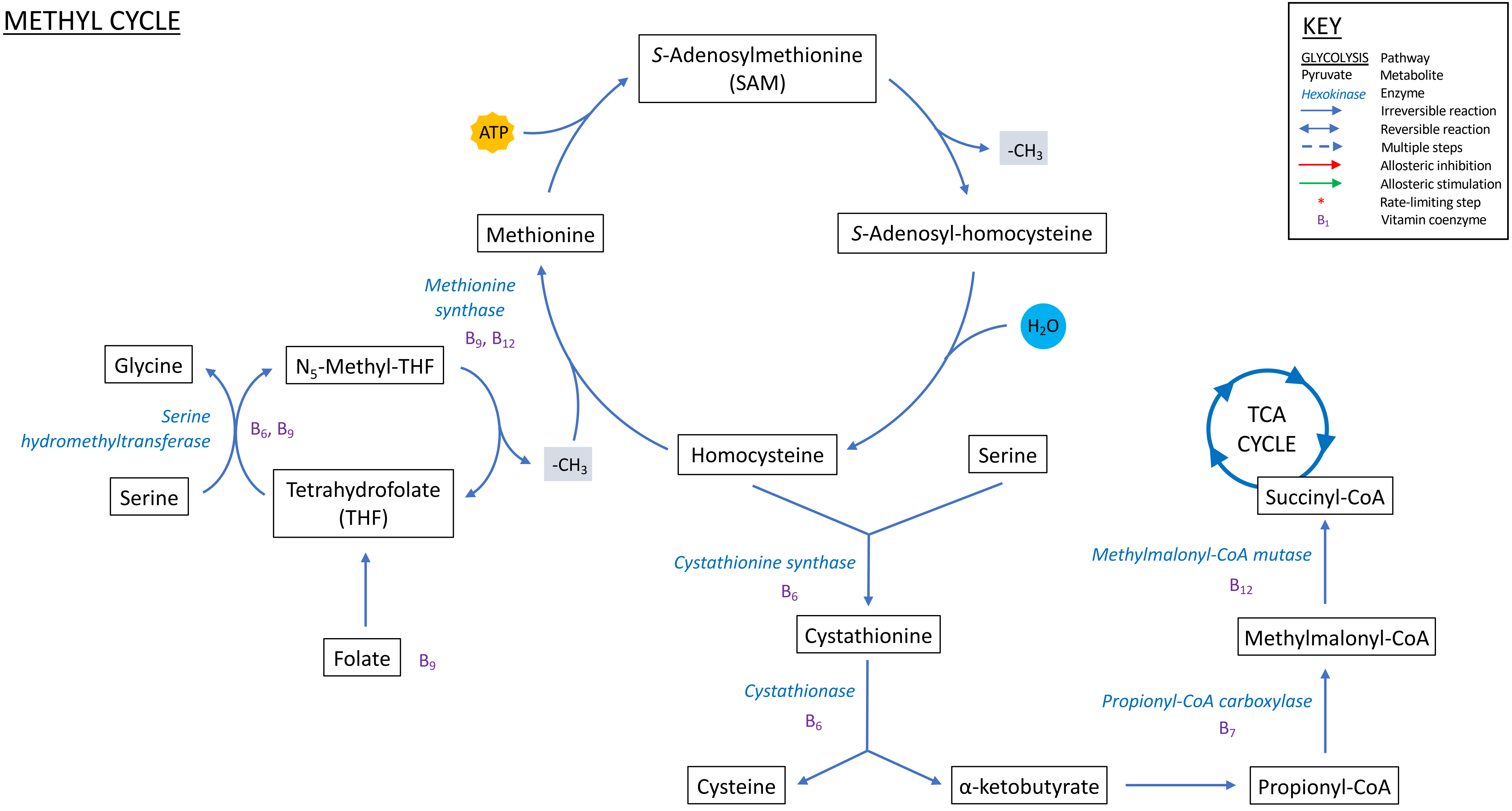

# METHYL CYCLE

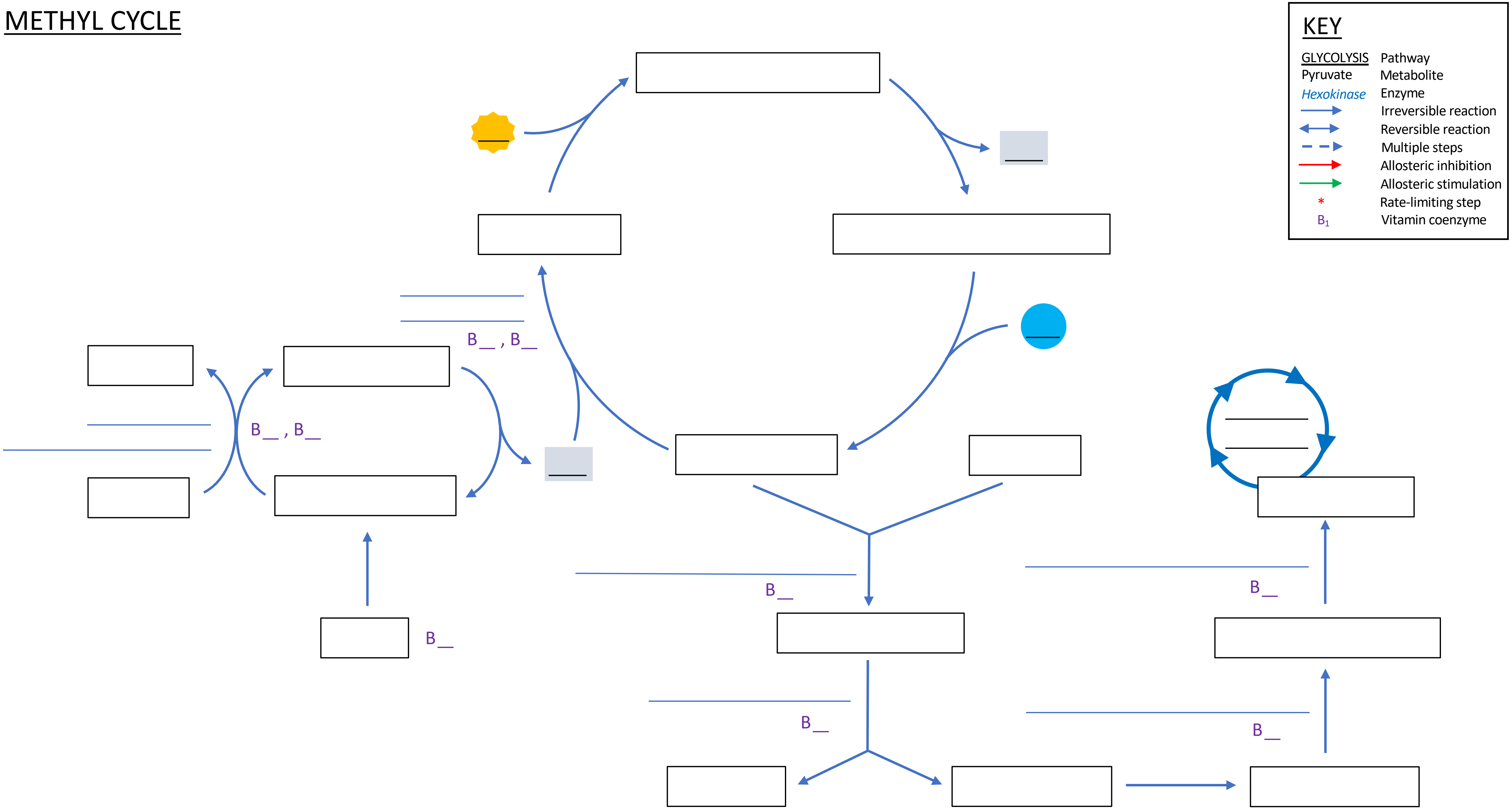

# UREA CYCLE

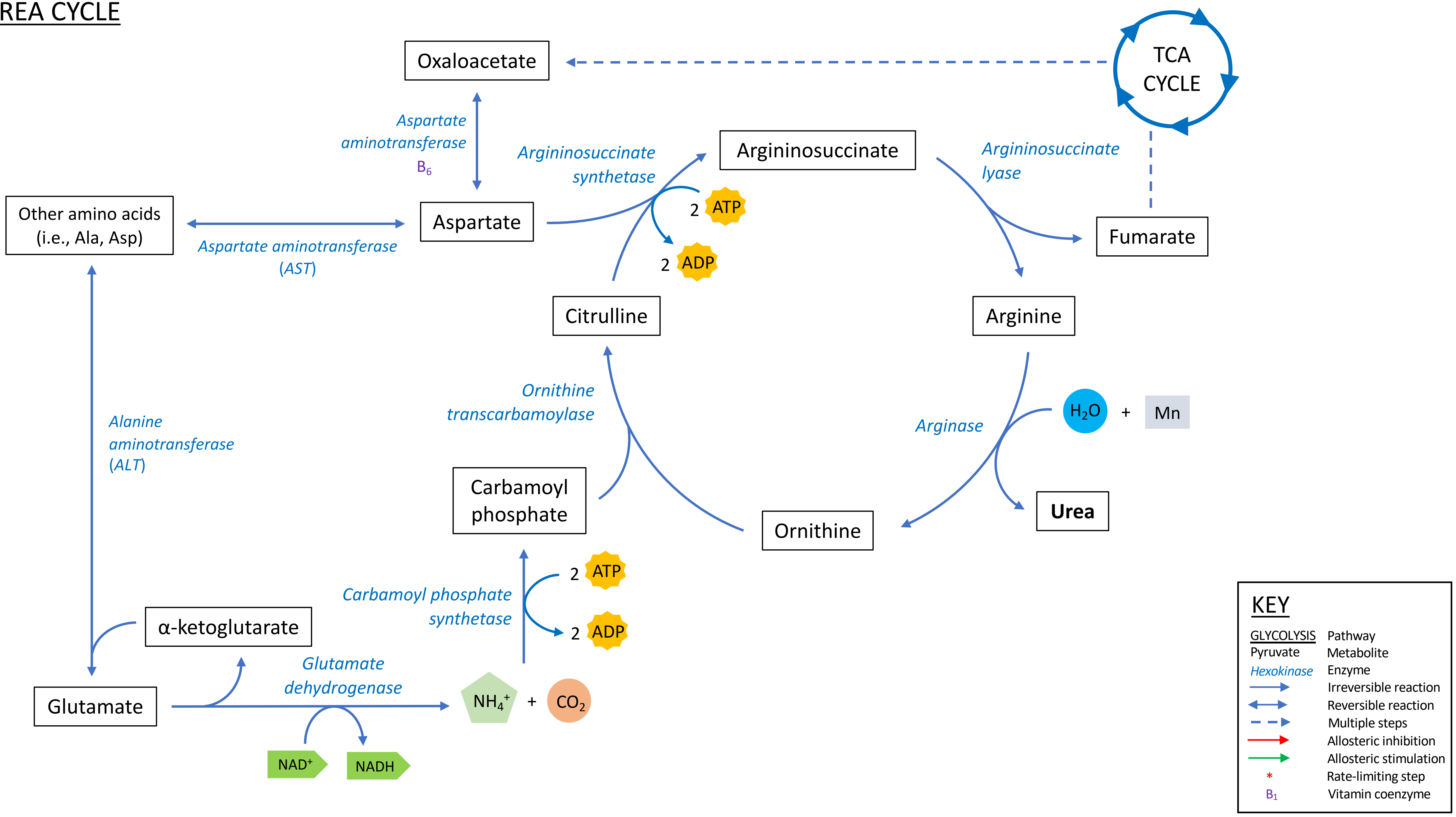

TCA CYCLE
Oxaloacetate
Aspartate aminotransferase
B6
Other amino acids (i.e., Ala, Asp)
Aspartate
Aspartate aminotransferase (AST)
Argininosuccinate synthetase
2 ATP
2 ADP
Argininosuccinate
Argininosuccinate lyase
Fumarate
Citrulline
Arginine
Ornithine transcarbamoylase
Arginase
H2O
+
Mn
Urea
Ornithine
Alanine aminotransferase (ALT)
Carbamoyl phosphate
Carbamoyl phosphate synthetase
2 ATP
2 ADP
α-ketoglutarate
Glutamate
Glutamate dehydrogenase
NH4+
+
CO2
NAD+
NADH
KEY
GLYCOLYSIS
Pathway
Pyruvate
Metabolite
Hexokinase
Enzyme
Irreversible reaction
Reversible reaction
Multiple steps
Allosteric inhibition
Allosteric stimulation
*
Rate-limiting step
B1
Vitamin coenzyme

# UREA CYCLE

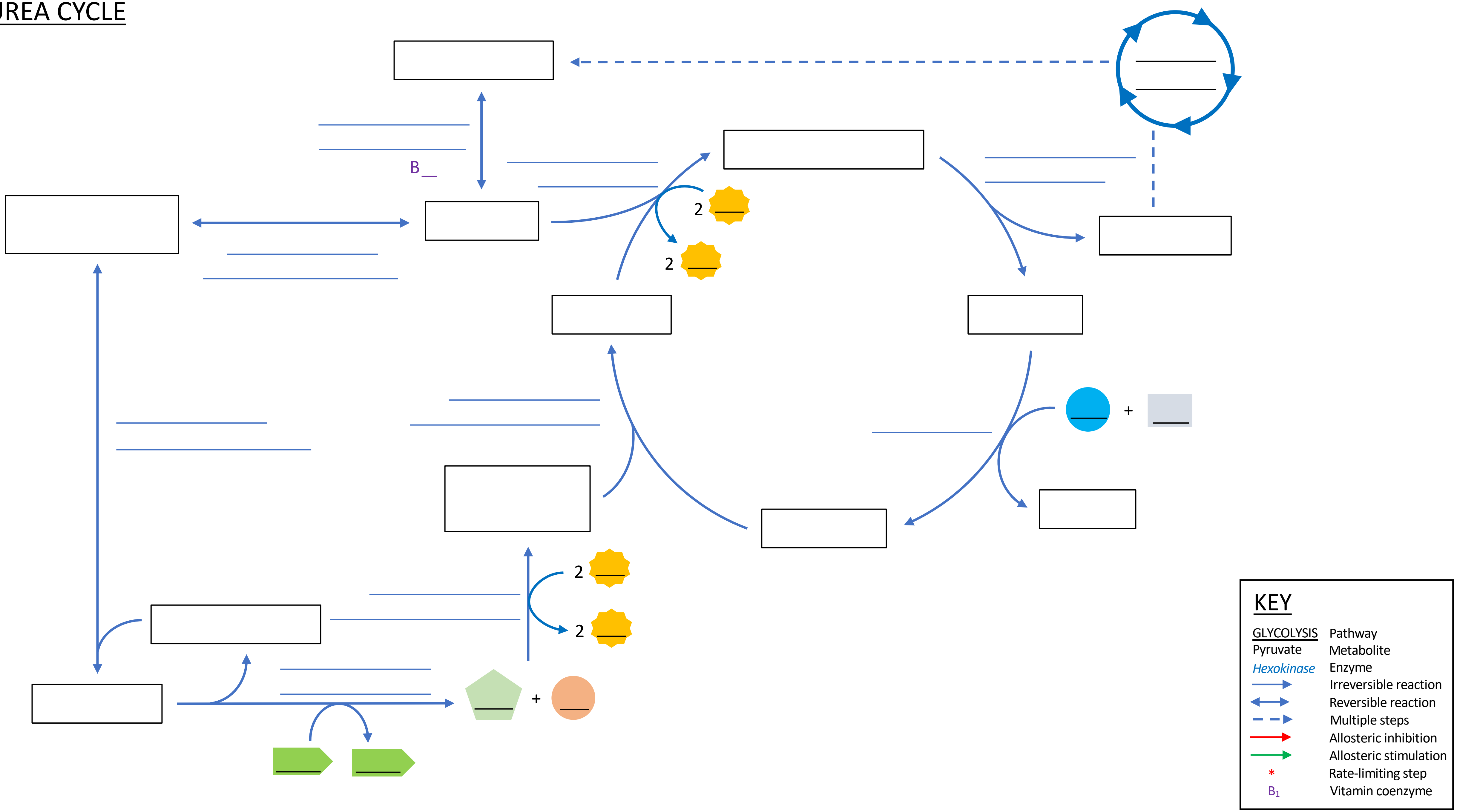
B__
2
2
2
2
+
+
KEY
GLYCOLYSIS Pathway
Pyruvate Metabolite
Hexokinase Enzyme
Irreversible reaction
Reversible reaction
Multiple steps
Allosteric inhibition
Allosteric stimulation
* Rate-limiting step
B1 Vitamin coenzyme
