# Supplementary material for: Use of Integrated Metabolic Maps as a Framework for Teaching Biochemical Pathways in the Pre-clinical Medical Curriculum
Source: Med Sci Educ. 2024 May 29;34(4):815–21. doi: 10.1007/s40670-024-02073-1 (PMC11296978; doi:10.1007/s40670-024-02073-1)
Supplement: Supplementary file 3 — Supplementary file3 (PDF 93 KB) [file 40670_2024_2073_MOESM3_ESM.pdf]

Student Evaluation Questions
Supplementary Online Resource 3

Article title: Use of Integrated Metabolic Maps as a Framework for Teaching Biochemical Pathways in the Pre-Clinical Medical Curriculum

Journal name: Medical Science Educator

Author names: Kenny Nguyen, Jay R. Silveira, Karen M. Lounsbury

Affiliation and email address of corresponding author:
The Robert Larner, MD College of Medicine, University of Vermont, Burlington, VT
karen.lounsbury@med.uvm.edu

Student Standard Student Session Evaluation

1. Please rate the overall effectiveness of the session materials (e.g., alignment with objectives; organization; clarity and communication of content).

Scale 1-5, with 5 being best

2. Please rate the overall effectiveness of the facilitator (e.g., communicating content, stimulating interest and enthusiasm for the subject, promoting problem solving/application of knowledge, etc)

Scale 1-5, with 5 being best

3. Please provide feedback on the facilitator's teaching or any of their sessions (e.g., specific strengths, suggestions for improvement)

Specific Metabolic Maps Survey

Experience with Metabolic Maps during Foundations of Clinical Sciences

This special survey, developed by the Office of Medical Education, aims to evaluate the usefulness of Metabolic Maps as a curriculum tool to understand the student experience and to improve the curriculum for future students.

Hello!

Thank you for participating in this brief survey. We appreciate your help in evaluating and improving Foundations curriculum. This survey is intended for current students in the Class of 2027 and is optional.

There are 3 questions in this survey.

1. The metabolic maps had a positive impact on my ability to integrate information about metabolic pathways.

Strongly Disagree/Disagree/Neutral/Agree/Strongly Agree

2. I used the metabolic maps to help in studying for medical biochemistry content on exams.

Never, Rarely, Sometimes, Often, Always

3. Please comment on the benefits and/or disadvantages of using the metabolic maps as a learning tool.
